# Ambient Electromagnetic Radiation as a Predictor of Honey Bee (*Apis mellifera*) Traffic in Linear and Non-Linear Regression: Numerical Stability, Physical Time and Energy Efficiency

**DOI:** 10.3390/s23052584

**Published:** 2023-02-26

**Authors:** Vladimir A. Kulyukin, Daniel Coster, Anastasiia Tkachenko, Daniel Hornberger, Aleksey V. Kulyukin

**Affiliations:** 1 Department of Computer Science, Utah State University, Logan, UT 84322, USA; 2 Department of Mathematics and Statistics, Utah State University, Logan, UT 84322, USA

**Keywords:** precision apiculture, precision beekeeping, electromagnetic radiation, electronic beehive monitoring, machine learning, regression, grid search, power use, energy efficiency, apiary science

## Abstract

Since bee traffic is a contributing factor to hive health and electromagnetic radiation has a growing presence in the urban milieu, we investigate ambient electromagnetic radiation as a predictor of bee traffic in the hive’s vicinity in an urban environment. To that end, we built two multi-sensor stations and deployed them for four and a half months at a private apiary in Logan, UT, USA. to record ambient weather and electromagnetic radiation. We placed two non-invasive video loggers on two hives at the apiary to extract omnidirectional bee motion counts from videos. The time-aligned datasets were used to evaluate 200 linear and 3,703,200 non-linear (random forest and support vector machine) regressors to predict bee motion counts from time, weather, and electromagnetic radiation. In all regressors, electromagnetic radiation was as good a predictor of traffic as weather. Both weather and electromagnetic radiation were better predictors than time. On the 13,412 time-aligned weather, electromagnetic radiation, and bee traffic records, random forest regressors had higher maximum $R^{2}$ scores and resulted in more energy efficient parameterized grid searches. Both types of regressors were numerically stable.

## 1. Introduction

In 1981, Kirschvink and Gould [1] hypothesized that some animals (e.g., honey bees, pigeons, and sharks) may possess special organs, which they called “magnetoreceptors”, for detecting magnetic field variations. The researchers conjectured that the biogenic magnetite (Fe${}_{3}$O${}_{4}$) discovered in honey bees, pigeons, and shark embryos may play a key role in their magnetoreception. Multiple precision apiculture studies have attempted to provide supporting evidence for the magnetoreception hypothesis by showing that different biological and behavioural characteristics of honey bees (*Apis mellifera*) may be affected by radio frequency and electromagnetic fields (RF EMFs) [2]. Hives exposed to high-voltage transmission lines show increased motor activity, abnormal propolisation, smaller weight gain, queen loss, and poor winter survival [3]. They generate sounds of higher intensity and frequency (e.g., “worker piping”) multiple hours after the end of exposure to the pulsed EMFs generated by regular mobile phones [4]. Honey bee losses and navigational abilities of foragers may be related to anthropogenic magnetic fields and natural geomagnetic storms [5]. Exposure to high-frequency radio waves increases bee mortality under some conditions when field exposure is maximized [6]. Proximity to cell phone towers negatively impacts incoming and outgoing hive traffic in *Apis cerana* colonies. [7]. Mobile phone radiation may induce alterations in antioxidant enzyme activities and lipid peroxidation levels and cause DNA damage in the exposed larvae [8]. Such radiation significantly reduces the hatching ratio of adult queens whose larvae were exposed and may alter pupal development [9]. Extremely-low-frequency electromagnetic fields (ELF EMFs) from powerlines impair the cognitive and motor abilities of exposed honey bees [10], reduce their aversive learning, and increase aggression [11]. In combination with pesticides, high-frequency electromagnetic fields (HF EMFs) cause American foul brood, higher bee mortality, queen failures, and excessive drone brood and honey storage [12].

Due to the ever increasing dependency of many communities all over the world on electrical devices and wired and wireless communication, the growing presence of ambient electromagnetic radiation (EMR), for the time being at least, is inexorable [2,11], as are the continuing urban sprawl and concomitant disappearance of native habitats. Many apiarists have no choice but to keep their hives in urban environments with higher EMR levels [9] due to the increasing unavailability of spaces in close proximity untouched by residential or commercial development. Consequently, insomuch as bee traffic is a contributing factor to hive health [13] and EMR is a growing component of the urban milieu, it is reasonable to ask if ambient EMR can be used as a predictor of bee traffic in hive vicinity in urban environments and how its predictive power compares to those of time and weather. While the latter, unlike the former, have a long research history in precision apiculture (e.g., [14,15,16,17,18]), to our knowledge, this study is the first one to analyze the predictive power of ambient EMR in linear and non-linear regression models of bee traffic in hive vicinity at an urban apiary using completely non-invasive means: no structural modification of the hive, no forced exposure of bees to artificially induced EMFs, no removal of individual bees from monitored hives for laboratory inspections with subsequent insecticide, and no sensors or fiducials in or on bees. In addition, unlike the other precision apiculture and machine learning studies we reviewed for this article, our study appears to be the first one to analyze the grid search of non-linear regression models of bee traffic in terms of numerical stability, physical run time, and energy efficiency, which has broader implications for machine learning at large.

The specific objectives of our investigation were: (1) to acquire a large dataset of time-aligned records from replicable weather, ambient electromagnetic, and video bee traffic sensors at an urban apiary for one complete beekeeping season in northern Utah, U.S.A; (2) to evaluate relative contributions of time, weather, and ambient electromagnetic radiation as independent variables in linear and non-linear (random forest and support vector machine) regression models of bee traffic in hive vicinity; (3) to run parameterized grid searches to discover optimal value ranges for the random forest and support vector machine hyperparameters; (4) to compare the relative performance of hive-specific and model transfer regressors; and (5) to run the parameterized grid searches on four different hardware platforms to test for numerical stability and to estimate the relative power use rates of the random forest and support vector machine regressors.

The remainder of our article is organized as follows. In Section 2, we detail the materials and methods of our investigation. In Section 3, we summarize our results. In Section 4, we discuss our results in terms of accuracy, physical run time, and numerical stability, which elucidates our rationale for excluding deep learning models from our investigation. In Section 5, we present our conclusions. We moved all tables and plots referenced in the text into the appendix to make the reading flow smoother. When we say that a particular table or plot represents a general trend, we mean that the tables and plots we computed from the curated datasets (e.g., tables or plots for the other dependent or independent variables or months) in the supplementary materials showed the same or similar trend, which can be replicated from our datasets by third parties.

The supplementary materials include all our curated datasets for this investigation (CSV files), spread sheets with additional tables, replicable assembly instructions for our weather–EMR monitoring stations (see Section 2), and our data collection software used in the stations. The materials also include several short videos to illustrate some hardware and software aspects of BeePi, our video logging (vlogging) and analysis system, with which the readers, especially those who may be unfamiliar with our previous work on video analysis of bee traffic in hive vicinity (e.g., [19,20,21]), may want to familiarize themselves before proceeding to the remainder of the article.

## 2. Materials and Methods

We used only off-the-shelf sensors in our investigation and implemented all regressors and parameterized grid search tools with open source software libraries. All operating systems on which we executed our cross-validation, numerical stability, and power efficiency tests are also open source. We preferred open source solutions to commercial alternatives to ensure that our materials and methods can be broadly applied by researchers, practitioners, and citizen scientists to study the impacts of ambient electromagnetic radiation on honey bee traffic.

### 2.1. Data Acquisition

#### 2.1.1. Environment

The city of Logan (latitude 41.73698${}^{{}^{\circ}}$, longitude −111.833836${}^{{}^{\circ}}$, elevation ≈ 1358.5 m above sea level), where we conducted this investigation, is located in northern Utah, a state of western U.S.A. The Logan area experiences two nectar flows per year: the major nectar flow from mid-May to late June and the minor nectar flow from mid-August to mid-September. The primary nectar and pollen sources are private vegetable and flower gardens and orchards. Local apiarists hive new bee packages in late April or early May and harvest the honey by mid-September. Regardless of the bee race (Carniolan, Italian, Russian, etc.), many bee packages offered for sale by local suppliers come to Utah by trucks from queen breeders in California and Georgia, two states of the U.S.A. different from northern Utah in terms of climate: in particular, many areas in those states have no extended periods of sub-zero Celsius temperatures and snow.

During the beekeeping season (May–September), the temperature can be as low as +5 ${}^{{}^{\circ}}$C in late April and early May and as high as ≥30 ${}^{{}^{\circ}}$C in July and August with a drop to +10 ${}^{{}^{\circ}}$C in September. In October, the temperature stays slightly above 0 ${}^{{}^{\circ}}$C with occasional, slight sub-zero dips; November brings in temperatures mostly below 0 ${}^{{}^{\circ}}$C with cold rains and snow showers. Snow stays on the ground from late November to early or mid-March; during this period, the temperature mostly hovers in the −5 ${}^{{}^{\circ}}$C to −10 ${}^{{}^{\circ}}$C range but may occasionally drop to ≤−20 ${}^{{}^{\circ}}$C. From December to February, the weather can be erratic, with the temperature leaping to +5 ${}^{{}^{\circ}}$C on one day making snowfall turn into rain and on the same or next day dropping to −10 ${}^{{}^{\circ}}$C with the rain turning back into snowfall. The winter air quality frequently worsens due to air inversions. The summer air quality may become abnormal in July and August due to forest fires in the geographically proximal states of Idaho, Oregon, and California.

#### 2.1.2. Weather and Electromagnetic Radiation Sensors

**Figure 1:**
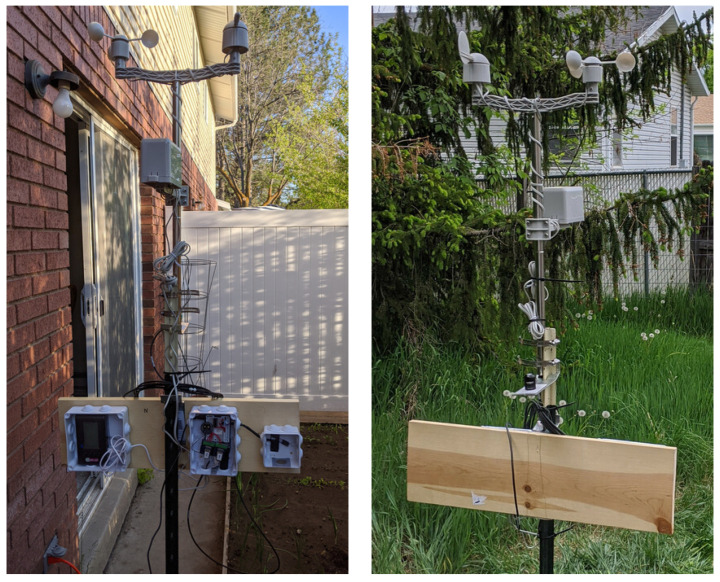
The left picture shows a weather station during a trial run in Logan in a small backyard next to a brick wall with a window; no data were collected at this site. The right picture shows the back side of the same station deployed at a private 6-hive apiary in Logan, where the weather and electromagnetic radiation data were collected from May 16 to 1 September 2020 (see Figure 2).

**Figure 2:**
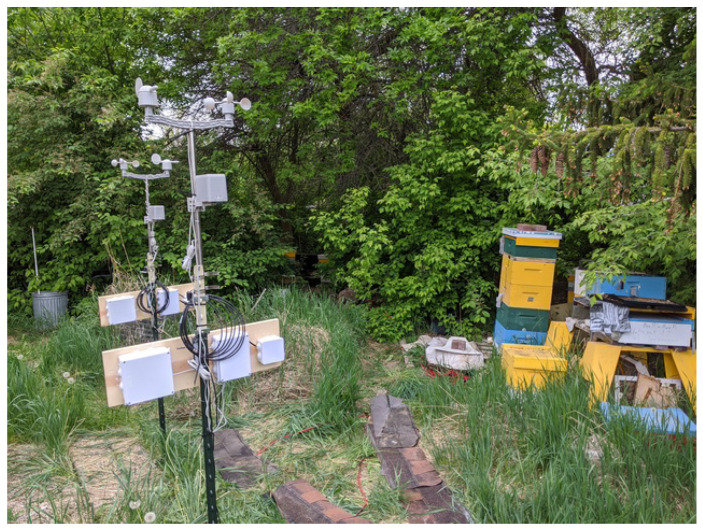
The two weather–EMR stations at a private apiary in Logan; a tower of four colored boxes on the right side of the picture is the closest Langstroth hive (≈7 m from the farthest station) with a live *Apis mellifera* colony; bottom to top, the hive consists of a light blue box, a green box, two yellow boxes, a telescoping hive lid, and a brick placed on top of the lid to keep it in place in strong winds; in American beekeeper jargon, the boxes are sometimes called *supers*; in the closest station the waterproof boxes on the wooden plank have their covers securely attached to them; both stations were installed at the apiary on 15 May 2020.

We built two stations, one for data collection, the other for redundancy in case of software and hardware failures, and deployed them at a six-hive private apiary in Logan to record ambient weather and EMR data [22] (see Figure 1 and Figure 2). As shown in Figure 1, the sensors are attached to a vertical metallic post crowned with a horizontal plastic bar. An anemometer is on the left side of the horizontal bar (close to the wall). A wind vane is on the right side of the horizontal bar. The grey plastic box attached to the vertical metallic post ≈ 50 cm below the horizontal bar is a rain gauge. A pyronometer is attached to the metallic post ≈ 50 cm below the rain gauge. A horizontal wooden plank is attached to the metallic post ≈ 50 cm below the pyronometer. Left to right on the plank are an open plastic box with an EMF-390, an open plastic box with a Raspberry Pi computer, and an open plastic box with a BME-280. The boxes are waterproof, and, when deployed, waterproof plastic covers are attached to the boxes with screws to protect the two sensors and the computer against the elements (see Figure 2).

The apiary where both stations were deployed was in a 15 m × 18 m wooded backyard (see Figure 2) of a private house in a southwestern residential neighbourhood of Logan. The stations were placed 3 m apart. The trees were ≈5–7 m from the stations and the closest hive was ≈7 m from the stations. Both stations were powered around the clock with waterproof extension cords plugged into a power splitter connected to a standard electrical outlet in the wall of a barn located ≈10 m east of the stations. The extension cords were securely pinned to the ground with metallic water hose pins to prevent interference with human foot traffic.

The anemometer, the wind vane, and the rain gauge from the Argent Data Systems [23] measured wind speed, gust, and precipitation, respectively. The anemometer has three equally spaced arms with cups at the end of each. The cups are attached to a shaft with a magnet at the end. As the magnet turns, a reed switch opens and closes due to the magnetic field. At a wind speed of 1.492 mph (2.401 kmh), the switch closes once per second. The wind speed is calculated by counting the number of switches per second. The wind vane includes a vertical blade attached to a rod with a shaft. A magnet is attached at the end of the shaft, which is situated at the center of eight reed switches connected to resistors of different values. As the vane is turned by the wind, the orientation of the magnet on the shaft causes some switches to close. An external resistor is used as a voltage divider, and the output voltage is measured with an analogue-to-digital converter (ADC). Since each switch is connected to a unique resistor, the output voltage is unique for each of the 16 represented directions, each 22.5${}^{{}^{\circ}}$ apart. The tipping bucket rain gauge includes a rocker with two cups on each end. The rocker has a hinge connection at the center so only one cup at a time is open to the sky for catching rain. When the cup fills to 0.02794 cm of water, the rocker tips, the rain water runs out, and the other cup opens to the sky. Each time the bucket tips, a switch is triggered allowing the number of tips to be counted to estimate the rainfall.

BME280 [24] measured the humidity, pressure, and temperature. The sensor’s operating ranges are from 0 to 100% for relative humidity, 300 to 1100 hPa for atmospheric pressure, and −40 to +85 ${}^{{}^{\circ}}$C for temperature, with the corresponding accuracies of ±3%, ±1 hPa, and ±1 ${}^{{}^{\circ}}$C, respectively. Since the atmospheric pressure sensor was factory calibrated for readings at sea level, we recalibrated it for the Utah elevation. The pyranometer from Apogee Instruments [25] measured short-wave radiation (SW Rad). This silicon-cell sensor measures the 350–1100 nm portion of the solar spectrum of global SW Rad, which is approximately 80% of the total spectrum. As solar energy enters the sensor, a voltage is produced and fed into an analogue-to-digital converter (ADC) to report SW Rad in Watts per square meter (W/m${}^{2}$). Each millivolt produced is equivalent to 5 W/m${}^{2}$, and the sensor produces a max voltage of ≈250 mV to measure up to ≈1250 W/m${}^{2}$ of SW Rad. Since the SW Rad data might have been skewed by the tree shadows at the apiary, we supplemented the pyranometer’s SW Rad readings with the SW Rad readings from a nearby Utah Climate Center weather station located on the Utah State University (USU) campus ≈3.2 km away from the apiary, and ≈91.44 m higher in elevation. The station is located in a wide open space with no trees or tall buildings in the vicinity. The USU station’s readings were downloaded from the station’s website [26]. The EMF-390 sensor [27] monitored RF EMFs. The EMF portion of the sensor monitors in the *X*, *Y*, and *Z* axes with a range of 0 to ≈500 mG at a resolution of 0.1 per 1 mG. The EF portion ranges from 0 to 1000 volts per meter (V/m) at a resolution of 1 V/m, and is frequency-independent. The RF portion ranges from 0.2 mW/m${}^{2}$ to ≈9999 mW/m${}^{2}$ at a resolution of 0.01 mW/m${}^{2}$, and can measure frequencies up to 10 GHz. The EMF-390 sensor was housed in a water-proof plastic box next to the Pi computer and powered from one of the computer’s USB ports. The MCP3208 12-bit ADC [28] converted the analogue voltage representing the wind direction, and the SW Rad voltage from the pyranometer.

We used a Raspberry Pi 3 model B+ 64-bit computer with the Raspbian operating system (OS) as the station’s controller. The Pi 3 model B+ platform has multiple general-purpose input/output (GPIO) pins and up to four USB ports for connecting hardware components. All weather and EMR sensors were connected to the Pi computer via the GPIO pins or the USB ports. The Pi 3 B+ model has a built-in 802.11.b/g/n/ac wireless Local Area Network (LAN), which was used for local wireless data transfers. No internet or cloud computing was used for data storage or processing for the reasons expounded in Section 4 and Section 5. The ChronoDot 2.1 Real-Time Clock [29] was coupled to the Pi computer to timestamp collected weather and EMR data. The Pi computer saved the weather and EMR data every 15 min in comma separated values (CSV) files on an attached 32G USB storage device. The rainfall was reset to zero at 23:59 every 24 h.

#### 2.1.3. Honey Bee Traffic Sensors

The video bee traffic data for this investigation were collected from two hives in the apiary, which we will refer to as R45 and R411 (the original IDs from the digital logs). Both hives had Russian bee packages from Knight Family Honey (www.knightfamilyhoney.com (accessed on 20 February 2023)), a supplier in Orem, Utah, U.S.A. The packages were hived by the first author in two deep Langstroth supers each on 25 April 2020. The first author, a licensed Utah beekeeper, manually inspected the hives in loco once a month from May to October 2020. No abnormalities, i.e., queen failure, American or European foul brood, chalk brood, excessive Varroa levels, or swarms, were detected. No chemical treatments were applied to either hive. The second deep Langstroth super was added to each colony on 1 July 2020. The third Langstroth super was added on 1 August 2020.

Two BeePi video loggers (vloggers) [30,31,32], one per hive, were installed on the two hives on 16 May 2020. Each vlogger consisted of an 8-megapixel Pi camera connected to a Raspberry Pi 3 Model B+ computer with the Raspbian Operating System (OS). The Pi computer was connected to a ChronoDot 2.1 Real-Time Clock; the timestamped videos were saved on a 5 terabyte USB device coupled to the Pi computer. The camera was protected against the elements and securely attached to the front side of a shallow Langstroth super (with the computer, the clock, and the USB device inside the super) on top of the hive. The super with the hardware equipment was separated from the hive with a wooden inner hive cover nailed to the super’s bottom; a metallic mesh covered the small hole in the middle of the inner cover to prevent the bees from crawling into the super from inside the hive. Four small holes (≈2.5 cm in diameter) were drilled on each side of the hardware super to improve ventilation. The camera looked down on the hive’s landing pad. The volume of the space in front of the hive in the recorded videos was approximately 3 m × 3 m × 3 m. It is this space that we call *the hive’s vicinity*. The Pi computer was powered around the clock through a waterproof extension cord plugged into a cord splitter connected to the same electrical outlet in the wall of the nearby barn from which the weather–EMR stations were powered. The computer was set to record and save 30 s videos (format: MPEG-4 (MP4), resolution: 1080 × 1980 pixels, frames per second (fps): 24) every 15 min from 7:30 to 20:45 daily from 16 May to 30 September 2020, for a total of 53 videos per day. The vloggers did not have any hardware or software failures during the observation period. They continued to function flawlessly in the presence of a wasp nest in the R45 hardware super in July and an ant nest in the R411 hardware super in August, both of which were discovered and removed during the monthly hive inspections. No structural modifications of the hives (e.g., special tunnels for bee exit and entry or plastic boards on top of the landing pad) were made and no sensors or fiducials were placed in or on bees. No videos were recorded 7–19 June 2020, due to a failure of the electrical outlet in the barn, from which the stations and the vloggers were powered, and on 13–14 August 2020, when the first author accidentally damaged a power cord splitter connected to the barn’s outlet and had to replace it. Each video was processed in vivo with the BeePIV algorithm [20,21] on the computer OGP (see Appendix A Table A1 and Table A2 for the computer’s software and hardware characteristics) to obtain the directional and omnidirectional bee motion counts for each video. BeePIV converts individual frames from videos to particle motion frames with uniform backgrounds and applies particle image velocimetry (PIV) methods [33] to each pair of consecutive motion frames to compute particle displacement vector fields. Depending on their directions, individual displacement vectors in the fields are classified as incoming, outgoing, and lateral. The total vector counts for each frame are used to measure incoming, outgoing, and lateral bee traffic in that frame. The sums of the three vector counts for all frames in a video give the counts of all incoming, outgoing, and lateral bee motion counts for the video. The application of BeePIV produced one CSV file that contained one row per every video with the upward (outgoing), downward (incoming), lateral, and total bee motion counts (non-negative integers) for the video.

Blackiston [34] estimates that “about 60,000 or more bees reside in a healthy hive”. According to Dadant [35], “in its usual working condition, a colony of bees contains a fertile queen, many thousands of workers, according to the season of the year, and in the busy season, from several hundred to a few thousand drones”. Thus, bee traffic in hive vicinity, which is the dependent variable (DNV) in all our models, consists mostly of foragers, but also includes drones.

Timestamps were used to align the bee motion counts from each video with the means of four consecutive weather and EMR measurements (three previous and one concurrent) and save the aligned records into two CSV files: one for R45 (6710 records) and one for R411 (6702 records). Only complete records were included in the merged CSV files: eight records were removed from the R411 data, because they were missing at least one of the weather or EMR readings. The timestamps in the final files were mapped to positive integers from 1 to 53 (e.g., 7:30 → 1, 8:00 → 2, …, 20:45 → 53). The two CSV files, R_4_5_s1_2020_DH.csv and R_4_11_s1_2020_DH.csv, are in the Supplementary Materials.

### 2.2. Data Analysis

#### Regression

The independent variables (INV) for all regressors were split into four categories: TIME, WEATHER, EMR, ALL, the latter being the union of TIME, EMR, WEATHER (see Table A3). Each regressor had exactly one DNV (see Table A4). Three types of regression models were evaluated: the linear regressor (LR), the random forest [36] regressor (RFR), and the support vector machine [37] regressor (SVMR). We use the notation INV→RGR→DNV to specify a regression model in terms of INV, DNV, and a regressor (RGR) (e.g., EMR→LR→COUT is a linear regressor whose independent variables are EMR and whose dependent variable is the cubic root of the outgoing bee motions). When a reference to a hive or month is required, the notation INV→LR→DNV{Hive,Month} is used (e.g., EMR→RFR→COUTPIN{R411,7} denotes a random forest regressor for the hive R411 and the month of July whose dependent variable is EMR and whose independent variable is the cubic root of the sum of the outgoing and incoming bee motions). Context permitting, we omit some elements from the notation for brevity (e.g., EMR→RFR→COUT, WEATHER→SVMR, LR→COUTPIN) or use the notation INV = TIME, INV = WEATHER, DNV = COUT, DNV = COUTPIN to denote models with specific independent and dependent variables.

The LR analysis was executed in R with 200 model types (see Table A5). For each month, Pearson’s correlation coefficients were computed between the EMF and WEATHER variables, between the EMF variables, and between the WEATHER variables. All model types were evaluated with a 70/30 train/test split. The non-linear regression (NLR) analysis was performed with the NLR model types, as shown in Table A5. RFRs and SVMRs were grid-searched with the 10-fold cross validation and the 70/30 train/test split to determine optimal hyperparameter ranges. We will refer to this NLR grid search as the *hive-specific grid search*. The NLR model hyperparameters and their ranges are in Table A6, Table A7 and Table A8. In the hive-specific grid search, for each 10-fold cross validation with the 70/30 train/test split, the absolute minimum and maximum $R^{2}$ (coefficient of determination) for each NLR model were recorded as well as the mean maximum $R^{2}$ and its standard deviation (STD). The analysis of the optimal hyperparameter values was performed on the top 30% of the NLR models ranked by the maximum $R^{2}$. To evaluate the NLR *model transfer* from hive to hive, the grid search was used to train all NLR models on the R45 data and test them on the R411 data, and, vice versa. We will refer to this grid search as the *model transfer grid search* to juxtapose it to the *hive-specific grid search*.

### 2.3. Numerical Stability, Physical Time and Power Use

The NLR models and the hive-specific and model transfer grid searches were implemented in Python with the numpy (www.numpy.org) and scikitlearn [38] libraries, two open source tools of the Python scientific computing stack. The control scripts were implemented in Perl. To ensure the numerical stability of the results, the Python and Perl programs were executed on four different computers (see Table A1 and Table A2). The physical run times (in seconds) were programmatically logged for each grid search on each computer and converted into hours for the subsequent power use (i.e., energy efficiency) analysis. We note in passing that we use the terms *power use* and *energy efficiency* synonymously. The power use data were taken from a Gardner Bender(TM) Power Meter PM3000. The meter was plugged into an electrical wall outlet and each of the three computers (OPC, PWE, EDW) were plugged into the meter to run for 24 h without running our grid search programs. The power use experiments were not executed on the computer OGP, because in December 2022, when we were completing the power use experiments, the computer was permanently damaged by a power outage at the Utah State University. After the 24 h period, the total cumulative power amount (CPA) in kilowatt-hours (kW-h) on the meter’s display was recorded. The meter was reset, and the RFR hive-specific grid search program was executed on the computer for another 24 h, and the CPA was recorded. After the meter was reset again, the SVMR hive-specific grid search program was executed on the computer for another 24 h, and the CPA recorded. The power use rates in each case were estimated as CPA/24. During all power use experiments, no other processes, except the regular background Linux OS processes, ran on the computer, the wireless and wired internet connections were disabled to prevent background updates, no USB devices were connected to the computer, and the computer’s monitor was turned off.

## 3. Results

### 3.1. Regression

Table A9 gives the Pearson’s correlation coefficients and the corresponding *p* values of (AVGEMF, TEMP), and (AVEMG, HUMID) (see Table A3 for the descriptions of AVGEMF, TEMP, HUMID). For all months, the (AVGEMF, TEMP) Pearson’s correlation coefficients and their *p* values were 0.95 (<0.0001); the (AVGEMF, HUMID) Pearson’s correlation coefficients and their *p* values were −0.82 (<0.0001). All other absolute values of the Pearson’s correlation coefficients were <0.7 or had *p* values > 0.05. Table A10 summarizes the LR results computed in R with the 70/30 train/test split. The lowest maximum $R^{2}$ of 0.10 was from TIME→LR→CIN{R45,5}; the highest maximum $R^{2}$ of 0.66 was from ALL→LR→COUTPIN{R411,9}. The (mean $R^{2}$, STD) of TIME→LR→DNV were (0.31, 0.12); of WEATHER→LR→DNV–(0.37, 0.12); of EMR→LR→DNV–(0.36, 0.12); and of ALL→LR→DNV–(0.45, 0.13).

The total number of the NLR models evaluated in the hive-specific grid search on the four computers (OGP, OPC, PWE, EDW—See Table A1 and Table A2) was (see Table A6, Table A7 and Table A8).
4 (number of computers) × 323,200 (number of RFR models) + 4 (number of computers) × (151,200 + 201,600) (SVMR models with non-polynomial (non-poly) and polynomial (poly) kernels) = 2,704,000.

The total number of the NLR models evaluated in the model transfer grid search was
2 (number of computers, i.e., PWE and EDW) × 323,200 (number of RFR models) + 1 (number of computers, i.e., EDW) × (151,200 + 201,600) (number of non-poly and poly kernel SVMR models) = 999,200.

Thus, all in all, the total number of the NLR models evaluated in the hive-specific and model transfer grid searches was
2,704,000 (hive-specific grid search models) + 999,200 (model transfer grid search models) = 3,703,200.

**Figure A1:**
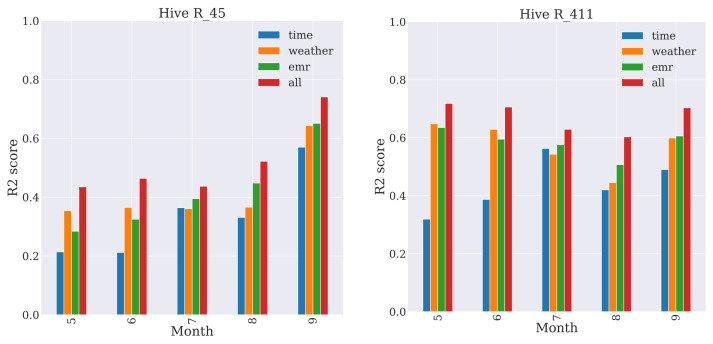

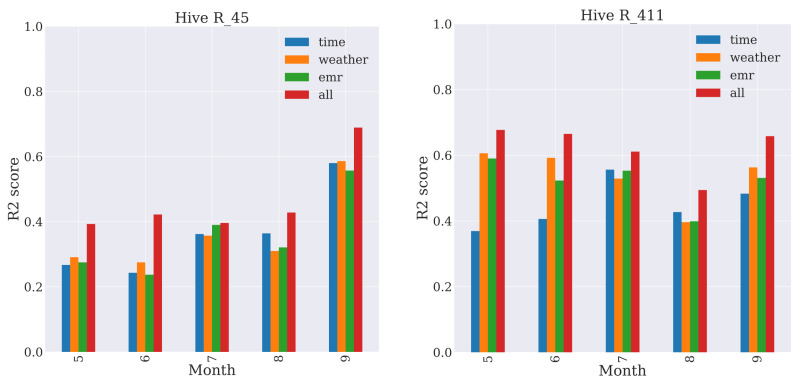
INV bar plots of maximum $R^{2}$ scores of INV→RGR→DNV{H,M} models; RGR is RFR (**top row**) and SVMR (**bottom row**); Hive (H) is hive R45 (**left column**) and hive R411 (**right column**); Month (M) is the *x*-axis; INV and DNV take on all possible values.

**Figure A2:**
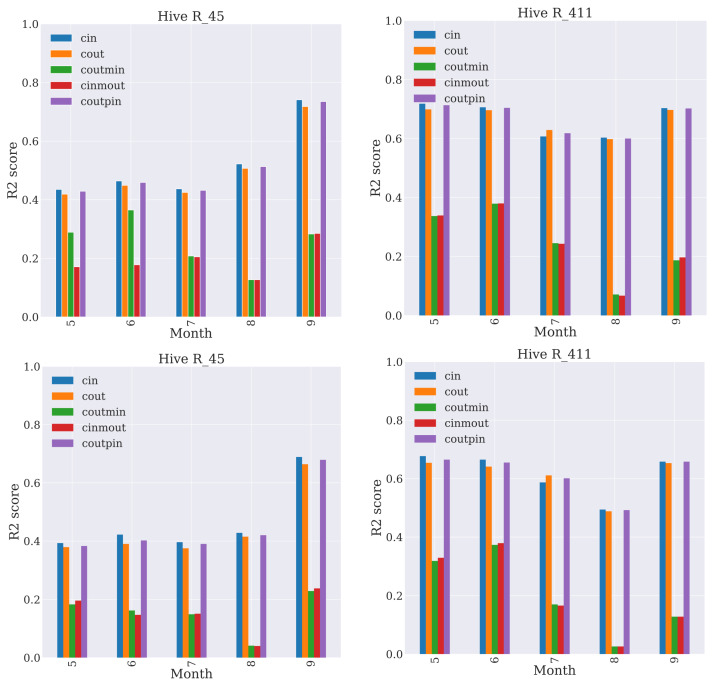
DNV bar plots of the maximum $R^{2}$ scores of INV→RGR→DNV{H,M} models; RGR is RFR (**top row**) and SVMR (**bottom row**); Hive (H) is hive R45 (**left column**) and R411 (**right column**); Month (M) is the *x*-axis; INV and DNV take on all possible values.

**Figure A3:**
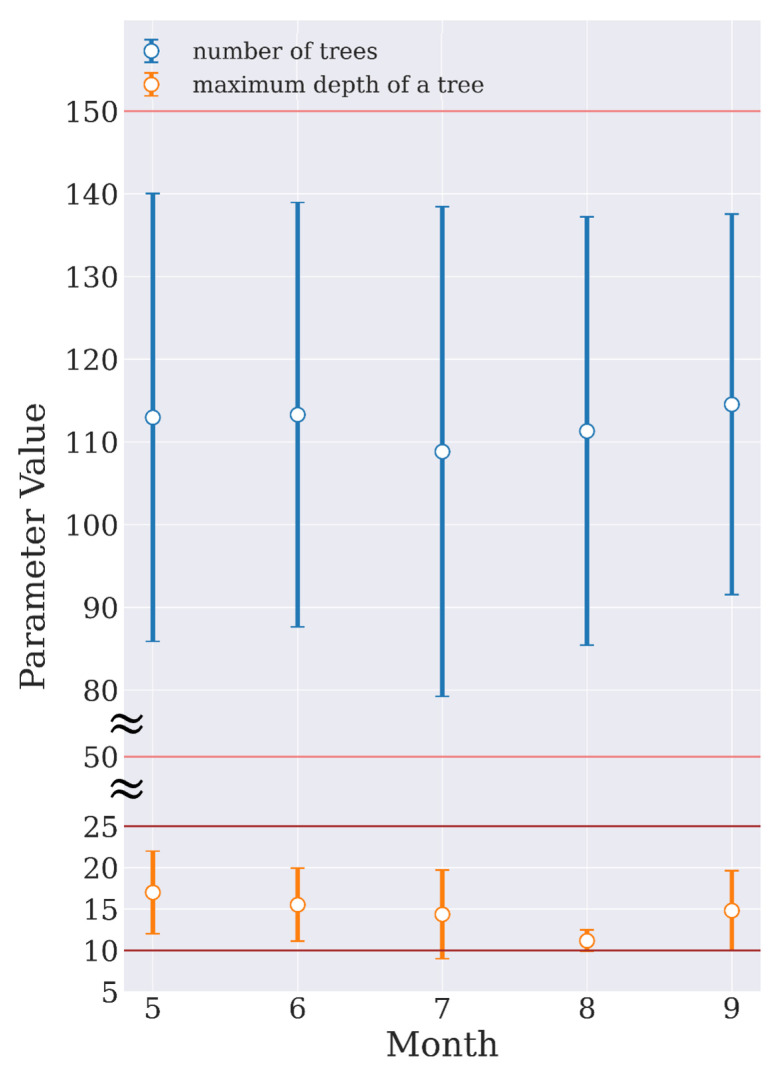
Mean value (dots) and corresponding standard deviation (vertical lines) of RFR hyperparameters (i.e., number of trees (NT)—blue and maximum tree depth—orange), of the top 30% (i.e., ranked by $R^{2}$) of RFR models found with 10-fold cross validation and 70/30 train/test split; upper and lower bounds of the number of trees—light red; upper and lower bounds of the maximum tree depth (MTD)—dark red; ≈ denotes removal of unused sub-ranges on the *y*-axis for compactness.

**Figure A4:**
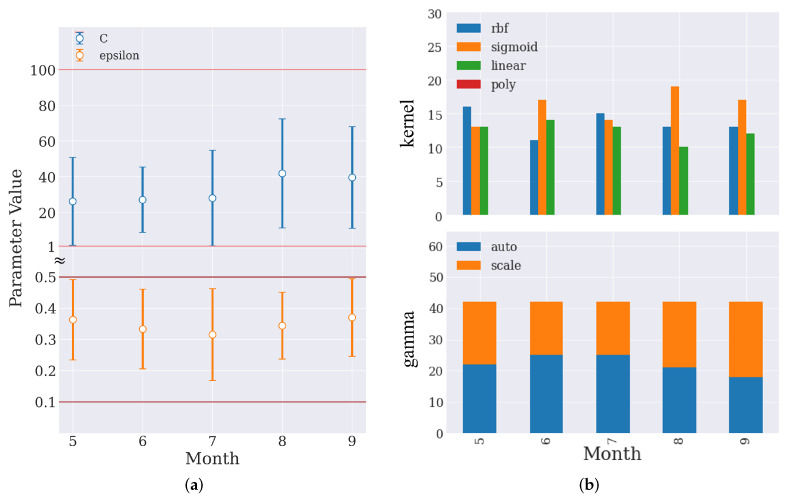
(**a**) Mean value (dots) and corresponding standard deviations (vertical lines) of SVMR hyperparameters (i.e., C—blue, epsilon—orange) of the top 30% (ranked by $R^{2}$) of SVMR models; upper and lower bounds of C—light red; bounds of epsilon—dark red; (**b**) frequency of *rbf*, *sigmoid*, *linear*, *poly* kernels (top); *auto* and *scale* for gamma hyperparameter (bottom).

**Figure A5:**
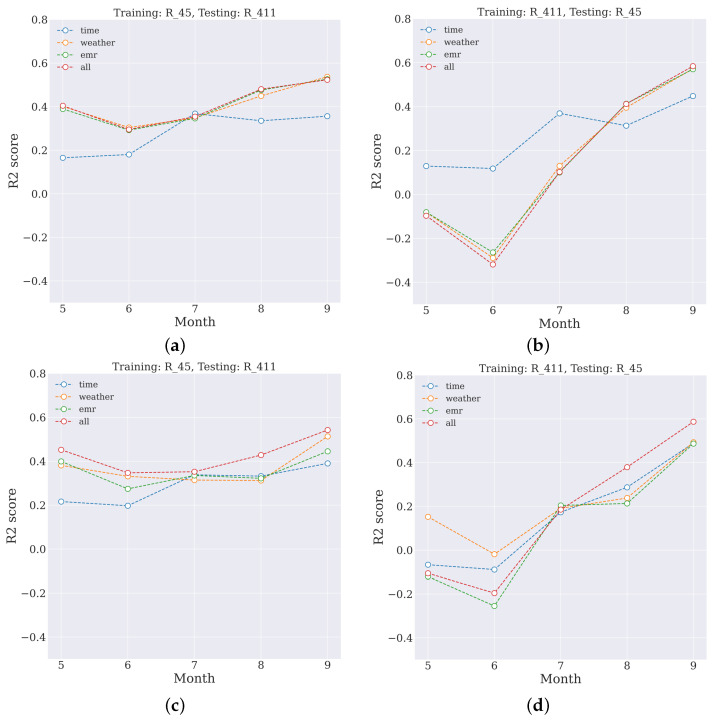
Mean of the maximum $R^{2}$ of INV→RFR→ DNV models (**a**,**b**) and INV→SVMR→DNV models (**c**,**d**) trained on R45 data and tested on R411 (**a**,**c**) and trained on R411 data and tested on R45 data (**b**,**d**).

Figure A1 and Figure A2 summarize the relative performance of the evaluated models with respect to INV and DNV in the hive-specific grid search. Figure A3 and Figure A4 and Table A11 summarize the hyperparameter statistics of the top 30% of the RFR and SVMR models ranked by the maximum $R^{2}$. The NT (number of trees) hyperparameter values of the top RFR models were in the chosen range of (50–150) (see Table A6); the MTD (maximum tree depth) hyperparameter values of the RFR models were in the range (10–25) in almost all models; in one model, the MTD was <10 in July. The hyperparameter values of the top SVMR models were all in the chosen ranges. The maximum $R^{2}$ scores of ALL→RGR→COUTPIN were on par with the maximum $R^{2}$ scores of all the other models for all regressors, hives, and months. The LR scores were the lowest for each month and hive; the SVMR scores were in the middle; the RFR were the highest (see Table A12). The performance for all other types of models showed the same comparative trends. Figure A5 summarizes the results of the model transfer grid search on the computer EDW.

### 3.2. Numerical Stability, Physical Time and Power Use

Table A13, Table A14, Table A15 and Table A21 summarize the numerical stability statistics for the two most structurally involved models: ALL→RFR→COUTPIN and ALL→SVMR→COUTPIN, respectively. The tables for all other models are omitted for brevity, because they exhibit the exact same numerical stability trends. Table A16 and Table A17 total the physical run times of the hive-specific grid search on four computers for all INV, DNV, hives, months, and computers; Table A18 and Table A19 do the same for the model transfer grid search; Table A20 summarizes the power use results.

## 4. Discussion

### 4.1. Regression

**Figure A6:**
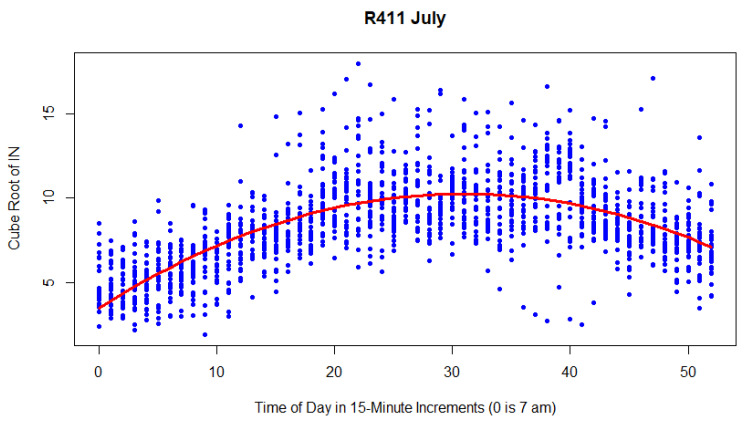
Scatter plot of CIN (cubic root of incoming bee motions) and quadratic regression for hive R411 for July.

Figure A6 shows the quadratic regression line of the cubic root of the incoming bee motion counts for all recorded times during the day for the R411 July data. The other hives and months showed similar traffic distributions. The quadratic regression lines showed better fits than the regular regression lines, which corroborates the findings of Marceau et al. [13] whose best regression model for their dataset of forager counts used quadratic regression. This observation also explains why we added quadratic effects to INV (see Table A3). Linear regression, in and of itself, was not sufficiently powerful to predict bee traffic patterns in the vicinity of either hive. Linear regression may not be sufficient to predict bee traffic in hive vicinity from electromagnetic radiation or weather, because traffic distributions, as Figure A6 demonstrates, do not appear to be linear. Nor do traffic distributions appear to be random. If they were random, scatter plots like the one in Figure A6 would not be seen, and  one would not see the $R^{2}$ scores of hive-specific models in the range of 0.30 to 0.70. Instead, one would expect to see the $R^{2}$ scores hovering at 0 or slightly above or be negative. More experiments are required to establish the exact extent of the non-randomness of hive-specific bee traffic in hive vicinity.

The INV = ALL NLR models performed better than the NLR models with INV = TIME, INV = WEATHER, or INV = EMR, which indicates that the components of bee traffic predicted by TIME, WEATHER, and EMR, by themselves, are not necessarily identical. Each INV category predicts its own traffic component and does not completely overlap with the components predicted by the other INV categories. The NLR models with INV = WEATHER predicted better in May and June and the models with INV = EMR—in July and August. However, the $R^{2}$ performance difference between the INV = EMR and INV = WEATHER models never exceeded 0.07. Thus, on our dataset and in our milieu, EMR and WEATHER were equally powerful as predictors and, therefore, interchangeable. The $R^{2}$ trend predicted by INV = TIME was the same for both hives and the predictive power of some models was higher in May and June, slipped in July and picked up in September, which closely coincides with the two major nectar flows in the Logan area (mid-May to late June; mid-August to mid-September). The forager traffic in Logan may become more predictable due to the availability of multiple nectar and pollen sources and less predictable when those sources are more difficult for forager scouts to locate. However, among all evaluated LR and NLR models, INV = TIME models performed the worst, which corroborates the findings of Polatto et al. [16] who concluded that “… time of day apparently had little influence on the foraging activity of the bees”.

The effects of WEATHER and EMR appear to have depended not on the NLR model type, but on the hive. The maximum $R^{2}$ scores tended to be almost identical for the INV = EMR and INV = WEATHER models for the same hive, but differed for the same model on different hives, which suggests that some bee traffic patterns may be unique to individual colonies. This result may corroborate the theory that a bee colony is a biological superorganism with its unique biotic characteristics [39]. In so far as the bee traffic reflects the unique biotic characteristics of the superorganism, to that extent it may be unique and vary from superorganism to superorganism.

The NLR models with DNV = CIN uniformally performed better than the models with DNV = COUT, DNV = CINMOUT, DNV = COUTMIN, and DNV = COUTPIN. However, the $R^{2}$ performance of these models differed by no more than 0.03, which makes DNV = CIN, DNV = COUT, and DNV = COUTPIN interchangeable. CIN and COUT may be preferred in the field, because they are faster to compute from videos than COUTPIN, the latter requiring computation of the cubic root sum of the incoming and outgoing bee motions instead of the cubic root of just one type of motion counts. Therefore, all things being equal, DNV = CIN/COUT models are expected to be more energy efficient on low power devices such as Raspberry Pi computers. The NLR DNV = CINMOUT and DNV = COUTMIN models performed worse than their counterparts with DNV = CIN/COUT/COUTPIN. The result was expected, because a 30-second time span of a single video is insufficient to capture the complexity of bee traffic. Since the nectar, pollen, and water sources are located at different distances from the hive, the foragers that leave the hive at the same time may not necessarily return to the hive at the same time and, conversely, the foragers that leave the hive at different times may come back at the same time. Both types of foragers will be captured by DNV = CIN, DNV = COUT, and DNV = COUTPIN but not necessarily by DNV = COUTMIN or DNV = CINMOUT. The dependent variables that estimate the difference between the incoming and outgoing bee traffic (such as CINMOUT and COUTMIN) behave more reliably for longer time spans (e.g., 3 to 6 h [21]).

Since the RFR hyperparameters NT and MTD were in the chosen ranges (see Table A6 and Figure A3) in the top performing models, the grid search ranges appear to have been chosen appropriately. In the RFR hive-specific grid search, the NT range can be made tighter by raising the lower bound to 75 and lowering the upper bound to 140, because all the top performing RFR models had between 75 and 140 decision trees. For the SVMR models, the upper bound of the hyperparameter C can be reduced to 80, because all top performing SVMRs had C ≤80. The range for the hyperparameter ϵ was chosen appropriately as well, because all top performing SVMRs had values of that hyperparameter in the specified range. The *linear* kernel occurred less frequently among the top SVMR models than the *rbf* or *sigmoid* kernels (see Figure A4), but had slightly higher mean maximum $R^{2}$ values (see Table A11). However, since the performance difference between these three kernels did not exceed 0.04, these kernels were interchangeable on our dataset. There were no *polynomial* kernel models among the top performers, which indicates that for our dataset, this kernel was not an adequate choice. Regarding the hyperparameter γ, only in June, the *auto* value of γ surpassed the *scale* value among the top 30% of the models, but the difference in the maximum $R^{2}$ was only 0.065. Overall, *auto* was preferable to *scale* insomuch as it was always either on par with or slightly better than *scale*.

Overall, RFRs performed better than SVMRs, which corroborates the robustness of random forests reported in other precision apiculture studies. Thus, Braga et al. [18] in investigating the problem of classifying the hive status from time and weather variables and manual hive inspections reported that the random forest classifiers outperformed the k-nearest neighbours, and neural networks. Kulyukin et al. [40] also reported that random-reinforced forests frequently performed on par with shallow convolutional networks in classifying bee images in videos. Although these investigations used random forests to solve classification, not regression, problems, they indicate that random forests remain viable alternatives to other machine learning models in precision apiculture. RFRs have a conceptual advantage over SVMRs in that they can, in a straightforward manner, be programmatically converted into symbolic descriptions in the form of sets of IF-THEN-ELSE-IF statements; SVMR hyperparameter interactions are less interpretable and therefore harder to analyze.

### 4.2. Model Transfer

**Figure A7:**
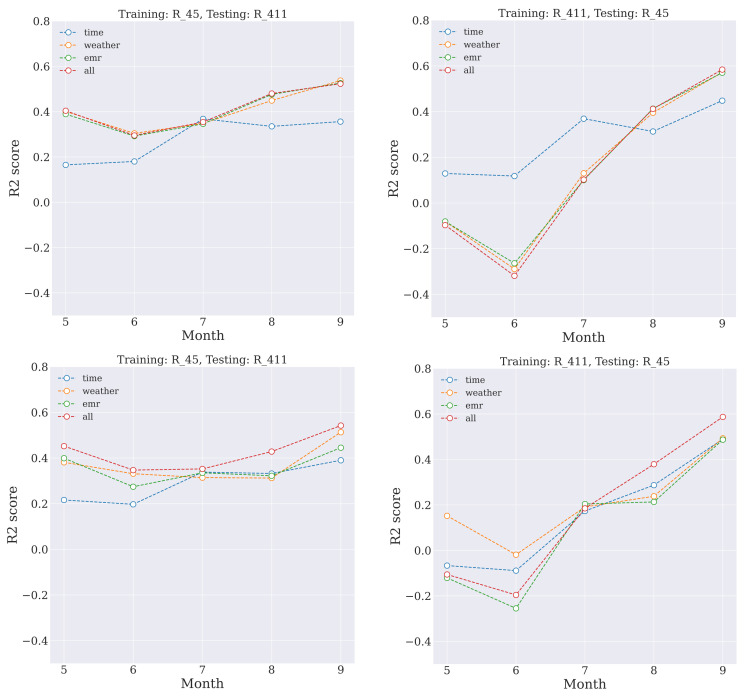
RFR and SVMR model transfer maximum $R^{2}$ score curves; RFR—(**top row**); SVMR—(**bottom row**); (**left column**): training on R45 data, testing on R411 data; (**right column**): training on R411 data, testing on R45 data.

The left column plots in Figure A7 show that the WEATHER/EMR/ALL→RFR models trained on the R45 data performed on par with each other; the $R^{2}$ scores of the TIME→RFR models were lower; for the WEATHER/EMR/ALL→SVMR models the $R^{2}$ scores fluctuated between 0.18 and 0.57 but had a wider spread than those of the WEATHER/EMR/ALL→RFR; the $R^{2}$ scores of the TIME→SVMR models were slightly higher than those of the TIME→RFR models. These observations suggest that between 18 and 57% of the bee traffic in the vicinity of the R411 hive could be predicted by the models transferred from the R45 hive. The lower performance of the INV = TIME models suggests that the time-dependent bee traffic patterns of the R45 hive did not coincide with the time-dependent patterns of the R411 hive. As the right column of Figure A7 indicates, the situation was reversed for the INV→RFR models trained on the R411 data and tested on the R45 data in that the TIME→RFR models performed better than the WEATHER/EMR/ALL→RFR models in May, June and July, with the $R^{2}$ scores fluctuating between 0.17 and 0.38; the scores of the WEATHER/EMR/ALL→RFR models for May and June were negative, which signals a complete lack of fit; the models recovered in July to 0.10 and then surpassed the TIME→RFR models in August and September, with the $R^{2}$ scores fluctuating between 0.4 and 0.58. The INV→SVMR models (bottom right graphs of Figure A7) had similar monthly trends with the rising $R^{2}$ scores in July, August and September; the only difference was that, unlike the TIME→RFR models, the TIME→SVMR models behaved on par with the WEATHER/EMR/ALL→SVMR models. Comparing the graphs in the top and bottom rows of Figure A7 suggests that R411 had more TIME-, WEATHER-, and EMR-dependent bee traffic in common with R45, than R45 had in common with R411. Overall, the model transfer $R^{2}$ scores of the NLR models were considerably lower than the $R^{2}$ scores of the hive-specific NLR models (See Figure A1 and Figure A2). The latter did not have any negative $R^{2}$ scores and many of them had $R^{2}$ above 0.60, which corroborates the earlier observation that bee traffic patterns may be hive-specific and differ from colony to colony and location to location.

### 4.3. Numerical Stability

In scientific computing, the results of a computation are considered numerically stable if multiple runs of the computation on the same software and hardware platform or on different platforms yield numerical results in the same ballpark. Numerical stability is a concern in numerical applications that deal with small and large reals or random numbers [41]. Consistent numerical results across multiple runs ensure not only consistency of accuracy but also consistency of error. As a quick example of numerical instability, let us consider the following interaction in Python 3.6.7 on a laptop with Ubuntu 18.04 (x86_64, 4 CPUs, Intel(R) Core(TM) i3-7100U CPU @ 2.40GHz, CPU max MHz = 897.051, BogoMIPS = 4800).

>>> import numpy as np
>>> x = 1e-308
>>> interval = np.linspace(0, x, 10)
>>> y1,y2 = interval[1],interval[2]
>>> (y1,y2)
(1.11111111111111e-309,2.22222222222222e-309)
>>> 0 < y1 and 0 < y2 and y1+1 == 1 and y2+1 == 1
True
>>> [0 < y < 1 and y + 1 == 1 for y in interval[1:]]
[True, True, True, True, True, True, True, True, True]

The above interaction is a constructive proof that in Python 3.6.7, the set of reals {y|y>0∧y+1=1}≠∅. Unfortunately, this interaction is not specific to a particular platform. With slight modifications in the printed output, it is replicable on all the computers in our study with different hardware architectures, flavours of Linux, and versions of Python and Perl. The interaction is also replicable in Python 2.7.17, which some scientific computing and numerical analysis researchers worldwide still consider the most numerically stable version of Python. We do not want to single out Python 2, Python 3, or the numpy library, which we, along with many fellow researchers from multiple disciplines, consider to be invaluable programmatic research tools, without which our study would have been impossible. Similar numerical instability instances are easily found in all the programming languages that our research group uses (C, Perl, Python). By Church’s thesis, they must exist in all programming languages. Our point is rather that, since numerical instability is a fact in many applications that use real numbers or random number generators, the results are likely more numerically stable if they do not greatly fluctuate in multiple runs on multiple platforms. In that regard, all NLR models evaluated in our study were numerically stable in that their $R^{2}$ scores did not fluctuate widely across the different computers, operating systems, and versions of Python, Perl, numpy, and scikitlearn. No instances were observed when the scores differed by 0.1 or higher; in a few instances the SVMR model scores differed by 0.07, which was expected, because the SVMR models had many more hyperparameters than their RFR counterparts.

### 4.4. Physical Run Time and Power Use

The hive-specific grid search took 1022.17 h for the RFR models and 1366.32 h for the SVMR models (see Table A16 and Table A17). These numbers, in and of themselves, may not mean much until we start to consider the amount of energy required to complete these grid searches (see Table A20). Using the mean power use rate estimates for the computers running the RFR and SVMR grid searches in the columns (COMP + RFR)/24 and (COMP + SVMR)/24, the RFR grid search is estimated to have taken 81.77 kW-h (i.e., 0.08 kW-h/h × 1022.17 h = 81.77 kW-h) and the SVMR grid search to have taken 95.64 kW-h (i.e, 0.07 kW-h/h × 1366.32 h = 95.64 kW-h), with a gain of 13.87 kW-h in favour of the RFR grid search.

How do these energy amounts compare to deep learning? In 2022, we trained a YOLOv3 network [42] to recognize individual bees in videos for our ongoing research on bee traffic video analysis. The YOLOv3 network was trained on a GTX 980 GPU computer running Ubuntu 18.04 LTS. The training was performed with the darknet system [43] compiled from its C source code and took ≈2500 h to achieve an average validation loss of 0.08. The power use rate of the GTX 980 computer running the network training program obtained from the same Gardner Bender(TM) Power Meter PM3000 was 0.24 kW-h/h for a total estimated amount of 600 kW-h (i.e., 0.24 kW-h/h × 2500 h = 600 kW-h).

According to the records available to us, a three-bedroom apartment with four residents in Logan used 491 kW-h in December 2019 and 382 kW-h in November 2017. If we multiply the estimated mean RFR energy use rate (0.08 kW-h/h in Table A20) by the number of hours in December (i.e., 31 × 24 = 744 h) and November (i.e., 30 × 24 = 720 h), we obtain 59.52 kW-h for December and 57.6 kW-h for November. Thus, the RFR search used ≈12% of the apartment’s energy amount in December 2019 and 15% of its energy amount in November 2017. The same calculation with the mean SVMR power rate of 0.07 kW-h/h (see Table A20) yields 52.1 kW-h for December (≈11% of the apartment’s energy amount in December 2019) and 50.4 kW-h for November (13% of the apartment’s energy amount in November 2017). The estimate with the combined RFR and SVMR mean power use rate of 0.15 kW-h/h (i.e., the sum of the two mean rates = 0.08 + 0.07) obtains 111.60 kW-h for December (23% of the apartment’s energy amount in December 2019) and 108 kW-h for November (28% of the apartment’s energy amount in November 2017). If we use the GTX 980 computer power use rate of 0.24 kW-h/h, we estimate the December energy amount at 178.56 kW-h (0.24 × 744 = 178.56 = 36% of the apartment’s energy amount in December 2019) and the November energy amount at 172.80 kW-h (0.24 × 720 = 172.80 = 45% of the apartment’s energy amount in November 2019). Machine learning has significant energy costs [44,45].

While there are numerous best practices and case studies (e.g., [18,36]), machine learning, both standard and deep, is hindered by a lack of mathematical theories that enable researchers to decide a priori (i.e., deductively, without running any experiments) which models will be best on a given dataset. The theorems by Wolpert and Macready [46,47] indicate that it may not be possible to determine a priori which models will perform best on a given problem or transfer best to another problem. To put it differently, there is no alternative to the actual experiment. Consequently, so long as there are no such theories, the constrained, parameterized grid search will remain the sole principled (as opposed to ad hoc) method to generate optimal, domain-specific predictive models. An immediate corollary of the last conclusion is that the concomitant considerations regarding numerical stability and energy efficiency use will necessarily apply.

This need for increasing amounts of power to search for optimal parameters, as we argued above and supported with calculations, has measurable costs associated with the required energy amounts and depletion of natural resources, such as water. Other research groups have recently started coming to the same conclusion that, in order to be cost effective, beehive monitoring machine learning models must operate in real time on low power hardware platforms such as the Raspberry Pi platform [48]. Therefore, in our future work, we will continue our search for machine learning models, methods, and algorithms for non-invasive precision apiculture that can operate on low power devices with limited or no access to cloud computing or the Internet.

Another hidden variable rarely discussed in the machine learning literature is the environmental costs of cloud computing extensively used for training models on various datasets. From June 2021 to May 2022, the top four data center owners in Utah alone consumed 149.8 million gallons of culinary water for cooling their computer facilities: NSA—128.3 million, Facebook—13.5 million, C7—6.9 million, and Novva—1.1 million [49]. The culinary water consumption for data centers is likely much higher, because Utah has many smaller data centers not considered in this article. In the meantime, as the historic drought continues in Utah, the Great Salt Lake, already at historically low levels as of September 2022, continues to lose water, with the Utah alfalfa farmers bearing the blame for consuming too much water [50].

### 4.5. EMR Impact on Honey Bees

A diligent, impartial reviewer of the precision apiculture literature on the effects of EMR on honey bees cannot but conclude that the evidence is controversial and inconsistent: some studies report negative impacts, while other studies report mixed results or no impact. Broadly speaking, the studies we found in the precision apiculture literature can be divided into two broad categories: non-invasive and invasive. The investigations in the former category (e.g., [7,12]) study the effects of ambient EMR on various aspects of honey bee colonies by measuring ambient EMR with sensors without any structural modifications of hives, placement of sensors on bees, or placement of EMR sources (e.g., smartphones) directly into hives. The latter category (e.g., [5,8,9,10,11]) includes studies that modify the hive or the bee, introduce EMR into the habitat, or expose individual bees extracted from a hive to artificially induced electromagnetic fields in the laboratory.

Lupi et al. [12], after a year-long investigation of the combined effects of pesticides and low- and high-frequency EMFs, reported the presence of American foul brood, higher mortality rates, queen changes, excessive drone brood and honey storage, with only one hive (out of four) surviving at a multi-stress site. The main findings by Shepherd et al. [10,11] were also negative in that the transient exposure to EMF was reported to reduce individual bees’ learning abilities, to impact their flight and foraging behaviours, to reduce aversive learning, and to increase aggression levels. However, all testing was performed with individual bees removed from a single hive and exposed to artificially induced radiation levels from a custom-built device in a laboratory. In the study by Ferrari [5], magnetized wires were glued to the abdomens of selected foragers to expose them to artificially induced fluctuating magnetic fields; the return rates of treated and untreated foragers released at different distances from their hives showed significant differences; correlations were found between forager loss and the Earth’s magnetosphere. The study by Darney et al. [6], on the other hand, reported mixed results: the exposure of honey bees to high-frequency radio waves increased mortality only in one condition (out of five): when the bees were exposed to high-frequency radio waves for 2 h per day; no negative effects on mortality were observed in the four other conditions. Odemer and Odemer [9] reported mobile phone radiation to reduce the hatching rate of queens whose larvae were exposed; however, no effects were observed on colony development if the treated queens successfully mated. Studies that report no negative effects also exist. For example, the investigation by Mall and Kumar [51] found no impact of EMR on brood rearing, honey production, or foraging behaviour of *Apis mellifera* L. colonies.

We agree with the observation of Odemer and Odemer [9] in their review of numerous studies of EMR effects on honey bees that “all examined studies were characterized by substantial shortcomings which were sometimes even admitted by their authors upfront”. We came to the same conclusion after our own review of the relevant literature. For example, in Favre’s investigation [4], a mobile phone (900 MHz; energy absorption rate < 2 W/kg) placed in a hive to play a radio station for several hours was observed to induce worker piping sounds in the hive. However, as Darney et al. [6] point out in their analysis of Favre’s results, one cannot rule out the possibility that the airborne sound signals perceived by honey bees induced the response [52]. That said, Favre’s investigation is thorough in describing the executed field study, especially the details of the hardware installation inside the hive and the measurement methodology.

As to the effects of ambient EMR on the two bee colonies in our study, both colonies at the beginning, during, and at the end of the monitored period were queenright. No abnormalities were detected during the monthly hive inspections. Both colonies had similar amounts of capped and uncapped honey, brood, larvae, and pollen at the end of the period. Thus, while in most LR and NLR models, EMR was as good a predictor as WEATHER and sometimes a better predictor than WEATHER and always a better predictor than TIME, we cannot report any observed impact of ambient EMR on the actual health of the two monitored colonies. Our investigation belongs in the non-invasive category of precision apiculture studies. We hope that our findings will be helpful to researchers and practitioners who seek non-invasive methods to study the effects of ambient EMR on honey bee colonies.

## 5. Conclusions

We caution that our conclusions are applicable only to datasets and environments sufficiently similar to ours (e.g., same or similar statistics and distributions of independent and dependent variables) and acknowledge that, given the great variability of bee races, climates and hardware sensors, broad generalizations are difficult. It may be the case that abiotic factors alone such as weather and electromagnetic radiation are insufficient to predict bee traffic completely [16]. More research is required to establish whether bee traffic patterns are unique to individual colonies and the statistically significant extent of that uniqueness.
**Conclusion 1:** Optimal hive-specific RFRs were found with the hyperparameter ranges in Table A6; the number of decision trees can be narrowed to the interval (75–140); the $R^{2}$ scores of optimal hive-specific RFRs were higher than those of optimal model transfer RFRs.
**Conclusion 2:** Optimal hive-specific SVMRs were found with the hyperparameter ranges in Table A7; the upper bound of the hyperparameter C can be lowered to 80; *linear*, *rbf* and *sigmoid* kernels were interchangeable; *poly* kernels performed worse than *linear*, *rbf* and *sigmoid* kernels; *auto* and *scale* values of the hyperparameter γ were interchangeable; the $R^{2}$ scores of optimal hive-specific SVMRs were higher than those of optimal model transfer SVMRs.
**Conclusion 3:** EMR and WEATHER were interchangeable as independent variables in LRs, RFRs and SVMRs and performed better than TIME; in applied research of bee traffic in urban environments, ambient EMR sensors may be used if and when WEATHER sensors are not available or in conjunction with the latter.
**Conclusion 4:** CIN, COUT and COUTPIN were interchangeable as dependent variables in LR, RFR and SVMR models; CINMOUT and COUTMIN performed worse than CIN, COUT and COUTPIN.
**Conclusion 5:** The parameterized grid searches of RFRs were more energy efficient than those of SVMRs.

## Data Availability

Details regarding where the data supporting the reported results can be found are given in the Supplementary Materials.

## Supplementary Materials

The following supporting information can be downloaded at: https://www.mdpi.com/article/10.3390/s23052584/s1, The document sensors_2195503_supmats.pdf in the supplementary materials gives a detailed description of each item.

## Abbreviations

The following abbreviations are used in this manuscript:

EMF	electromagnetic field
RF EMF	radio frequency and electromagnetic field
ELF	extremely low frequency
HF	high frequency
EMR	electromagnetic radiation
CSV	comma separated values
USB	universal serial bus
LAN	local area network
LTS	long-term support
vlogging, vlogger	video logging, video logger
fps	frames per second
SW	short-wave
SW Rad	short-wave radiation
ADC	analogue-to-digital
RFR	random forest regressor
SVMR	support vector machine regressor
RBF	radial basis function
LR	linear regressor/regression
NLR	non-linear regressor/regression
STD	standard deviation
INV	independent variable(s)
DNV	dependent variable(s)
NT	number of trees (a hyperparameter in RFR)
MTD	maximum tree depth (a hyperparameter in RFR)
CIN	cubic root of incoming bee motions
COUT	cubic root of outgoing bee motions
COUTPIN	cubic root of sum of incoming and outgoing bee motions
CINMOUT	cubic root of difference of incoming and outgoing bee motions
COUTMIN	cubic root of difference of outgoing and incoming bee motions
CPU	central processing unit
GPU	graphic processing unit
CPA	cumulative power amount
kW-h	kilowatt-hour
V/m	volts per meter
W/$m^{2}$	watts per square meter
W/kg	watts per kilogram
mG	milligauss
Hz	Hertz
MIPS	millions of instructions per second
BogoMIPS	Bogus MIPS, crude measurement of CPU speed by the Linux kernel when it boots to calibrate internal busy-loop
mph	miles per hour
kmh	kilometers per hour

## Appendix A

### Appendix A.1. Tables

**Table A1:** Software details of the four computers and hive-specific and model transfer NLR grid search programs; column 1 contains the computer IDs from the CSV files with complete experimental results in the Supplemental Materials; Foral Fossa is Ubuntu 20.04 LTS; Bionic Beaver is Ubuntu 18.04 LTS; GCC is Gnu C/C++ compiler.

Computer ID	Operating System	Python	Numpy	Sklearn	Perl
OGP	Foral Fossa	3.8.13 (GCC 7.5.0)	1.23.1	1.1.2	5.30.0
PWE	Bionic Beaver	3.6.9 (GCC 8.4.0)	1.19.5	0.24.2	5.26.1
OPC	Foral Fossa	3.8.10 (GCC 9.4.0)	1.23.4	1.1.3	5.30.0
EDW	Bionic Beaver	3.6.9 (GCC 8.4.0)	1.19.5	0.24.2	5.26.1

**Table A2:** Hardware details of the four computers on which hive-specific and model transfer NLR grid search programs were executed; column 1 contains the computer IDs in the CSV files with complete experimental results in Supplemental Materials.

Computer ID	Model	Arc	CPU	Num CPUs
OGP	GEFORCE RTX 2080 Ti	x86_64	i7-9700K@3.60 GHz	8
PWE	Dell PowerEdge T130	x86_64	E3-1230 v6@3.50 GHz	8
OPC	Dell Optiplex 9020	x86_64	i7-4770 CPU@3.40 GHz	8
EDW	HP Z240	x86_64	i7-6700 CPU@3.40 GHz	8

**Table A3:** Independent variables (INV) of the LR and NLR models; TIME models have 2 INV (variables 1,2); WEATHER models—8 (variables 3–10); EMR models—6 (variables 11–16; ALL models—16; W/m${}^{2}$—watts per square meter; mG—milligauss.

Num	INV Name	INV Category	INV Description
1	timept	TIME	time point, integer in [1, 53] (time of day)
2	timept2	TIME	timept${}^{2}$
3	pressure	WEATHER	atmospheric pressure (millibars)
4	pressure2	WEATHER	pressure${}^{2}$
5	humidity	WEATHER	relative humidity (percent)
6	humidity2	WEATHER	humidity${}^{2}$
7	windspeed	WEATHER	wind speed (miles per hour)
8	windspeed2	WEATHER	windspeed${}^{2}$
9	temp	WEATHER	ambient temperature (Fahrenheit)
10	temp2	WEATHER	temp${}^{2}$
11	swradusu	EMR	USU Climate Center short wave radiation (W/m${}^{2}$)
12	swradusu2	EMR	swradusu${}^{2}$
13	avgemf	EMR	mean strength of detected EMF (mG)
14	avgemf2	EMR	avgemf${}^{2}$
15	avgtotden	EMR	average total RF power flow per unit area (Watts/m${}^{2}$)
16	avgtotden2	EMR	avgtotdens${}^{2}$

**Table A4:** Dependent variables (DNV) of the LR and NLR models; IN—number (non-negative integer) of incoming bee motions; OUT—number (non-negative integer) of outgoing bee motions; (IN − OUT)—difference of IN and OUT; (OUT − IN)—difference of OUT and IN; (OUT + IN)—sum of OUT and IN.

Num	DNV Name	DNV Description
1	CIN	IN${}^{1/3}$
2	COUT	OUT${}^{1/3}$
3	COUTMIN	(OUT − IN)${}^{1/3}$ if OUT ≥ IN; −|OUT − IN|${}^{1/3}$ otherwise
4	CINMOUT	(IN − OUT)${}^{1/3}$ if IN ≥ OUT; −|IN − OUT|${}^{1/3}$ otherwise
5	COUTPIN	(OUT + IN)${}^{1/3}$

**Table A5:** Structural components of regression model types; RGR abbreviates “regressor”; LR—linear regressor; RFR—random forest regressor; SVMR—support vector machine regressor; total number of LR model types is 2×5×4×1×5=200, where 1 denotes LR; total number of NLR model types is 2×5×4×2×5=400, where 2 denotes RFR and SVMR.

Hive	Month	INV	RGR	DNV
R45	5	TIME	LR	CIN
R411	6	WEATHER	RFR	COUT
	7	EMR	SVMR	CINMOUT
	8	ALL		COUTMIN
	9			COUTPIN

**Table A6:** RFR hyperparameters; see online documentation of the scikitlearn library for details at www.scikitlearn.org; the total number of models in the hive-specific grid search is the number of hives × the number of months × the number of INV categories × the number of DNV × the number of values in the NT range × the number of values in the MTD range = 2×5×4×5×101×16 = 323,200 (see Table A5 for the number of structural components in RFRs).

Num	Name	Range	Description
1	NT	{i|50≤i≤150}	number of trees in RFR; 101 values
2	MTD	{j|10≤j≤25}	maximum tree depth in RFR; 16 values

**Table A7:** SVMR hyperparameters for the non-polynomial kernel models; the hyperparameter C controls the softness of the margin (the larger it is, the fewer points lie in the margin); the hyperparameter ϵ specifies the width of the tube within which no penalty is associated in the training loss function with points predicted within the distance of ϵ from the actual value; see online documentation at www.scikitlearn.org for more details; the total number of non-polynomial kernel SVMR models is the number of hives × the number of months × the number of INV categories × the number of DNV × the number of values in the C range × the number of values in the γ range × the number of values in the ϵ range × the number of kernels = 2×5×4×5×14×2×9×3 = 151,200. (See Table A5 for the number of structural components in SVMRs).

Num	Name	Range/Value
1	C	{1}∪{i+5|0≤i≤25}∪{i+10|30≤i≤90}; 14 values
2	γ	{scale, auto}
3	ϵ	{i+0.05|0.05≤i≤0.45}; 9 values
4	kernel	{linear, rbf, sigmoid}
5	cache_size	1000; this is just one value common to all models

**Table A8:** SVMR hyperparameters for the polynomial (poly) kernel models; the C, γ, ϵ ranges and cache_size value are the same as for the non-poly kernel models in Table A7; see online documentation at www.scikitlearn.org for more details; total number of polynomial kernel SVMR models is number of hives × number of months × number of INV categories × number of DNV categories × number of values in the C range × number of values in the γ range × number of values in the ϵ range × number of kernels × number of degree values = 2×5×4×5×14×2×9×1×4 = 201,600 (see Table A5 for the numbers of structural components in SVMRs).

Num	Name	Range/Value
1	kernel	poly; this is just one value common to all models
2	degree	[2, 3, 4, 5]; 4 values

**Table A9:** (Pearson, *p* value) between AVGEMF(A), TEMP(T), and AVGEMF(A), HUMID(H) for combined R45 and R411 data; Pearson’s, *p* values are rounded to two and four decimals, respectively; months are in columns; monthly number of observations (N) are: 774 (5); 1361 (6); 1578 (7); 1422 (8); 1575 (9); the Supplementary Materials include the spreadsheet ModelCVFits.xlsx which contains, under the tab “Correlations”, the tables of all Pearson’s of (EMR,INV) and (WEATHER,INV) and the corresponding *p* values.

	5	6	7	8	9
A,T	(0.97, <0.0001)	(0.96, <0.0001)	(0.93, <0.0001)	(0.97, <0.0001)	(0.93, <0.0001)
A,H	(−0.85, 0.0001)	(−0.89, <0.0001)	(−0.90, <0.0001)	(−0.89, <0.0001)	(−0.76, <0.0001)

**Table A10:** LR results with 70/30 train/test split; INV and DNV are in rows, months in columns; in entry x;y, $x\;=\;R^{2}$ for R45, $y\;=\;R^{2}$ for R411 for column month; lowest and highest $R^{2}$ values are bolded; all reals are rounded two decimals.

INV,DNV/Month	5	6	7	8	9
TIME,CIN	0.16; 0.31	**0.10**; 0.31	0.33; 0.50	0.22; 0.30	0.51; 0.40
TIME,COUT	0.19; 0.41	0.11; 0.28	0.29; 0.46	0.17; 0.26	0.50; 0.42
TIME,COUTPIN	0.20; 0.38	0.14; 0.36	0.29; 0.46	0.21; 0.22	0.50; 0.38
WEATHER,CIN	0.18; 0.42	0.16; 0.55	0.34; 0.43	0.23; 0.40	0.49; 0.46
WEATHER,COUT	0.19; 0.53	0.19; 0.51	0.32; 0.47	0.18; 0.34	0.45; 0.44
WEATHER,COUTPIN	0.27; 0.58	0.21; 0.52	0.29; 0.40	0.29; 0.32	0.48; 0.42
EMR,CIN	0.15; 0.42	0.16; 0.47	0.31; 0.42	0.28; 0.43	0.49; 0.39
EMR,COUT	0.23; 0.56	0.18; 0.46	0.29; 0.47	0.22; 0.40	0.49; 0.47
EMR,COUTPIN	0.22; 0.51	0.17; 0.45	0.29; 0.41	0.32; 0.34	0.51; 0.39
ALL,CIN	0.28; 0.48	0.32; 0.60	0.37; 0.56	0.41; 0.48	0.57; 0.50
ALL,COUT	0.30; 0.64	0.23; 0.55	0.35; 0.57	0.23; 0.44	0.57; 0.54
ALL,COUTPIN	0.36; **0.66**	0.28; 0.59	0.34; 0.49	0.37; 0.37	0.60; 0.48

**Table A11:** (MEAN,STD) of maximum $R^{2}$ in top 30% of INV→SVMR models; minimum and maximum $R^{2}$ means are bolded.

MONTH	RBF	SIGMOID	LINEAR	POLY	AUTO	SCALE
5	(0.537, 0.115)	(0.514, 0.120)	(0.542, 0.103)	nan	(0.542, 0.112)	(0.519, 0.112)
6	(0.531, 0.102)	(0.505, 0.104)	(0.544, 0.081)	nan	(0.551, 0.090)	(0.486, 0.094)
7	(0.536, 0.054)	(0.540, 0.019)	(0.555, 0.028)	nan	(0.547, 0.031)	(0.538, 0.046)
8	(0.428, 0.028)	(0.431, 0.038)	(**0.415**, 0.039)	nan	(0.426, 0.038)	(0.426, 0.033)
9	(0.594, 0.044)	(0.609, 0.054)	(**0.630**, 0.046)	nan	(0.605, 0.047)	(0.614, 0.052)

**Table A12:** ALL→RGR→COUTPIN, RGR is specified in columns; in entry (x;y), *x* is the mean $R^{2}$ for hive R45 and *y* is the mean $R^{2}$ for R411; highest $R^{2}$ values are bolded for each month and hive.

MONTH	LR R45; R411	RFR R45; R411	SVMR R45; R411
5	0.36; 0.66	**0.44; 0.72**	0.39; 0.68
6	0.28; 0.59	**0.46; 0.71**	0.42; 0.67
7	0.34; 0.49	**0.44; 0.63**	0.40; 0.61
8	0.37; 0.37	**0.52; 0.60**	0.43; 0.49
9	0.60; 0.48	**0.74; 0.70**	0.69; 0.66

**Table A13:** (MEAN, STD) of maximum $R^{2}$ of ALL→RFR→COUTPIN in the hive-specific grid search on computers OPC and PWE; computer IDs are names of columns 1,2; in cell is x;y|z;w, *x*, *y* are the mean max $R^{2}$ and its STD for hive R45, *z*, *w* are the mean max $R^{2}$ and its STD for hive R411; reals are rounded to two decimals; maximum and minimum $R^{2}$ are bolded; ties broken arbitrarily.

MONTH	OPC	PWE
5	**0.43**; 0.04 **0.71**; 0.03	**0.42**; 0.05 **0.71**; 0.03
6	0.46; 0.03 0.70; 0.02	0.46; 0.03 0.70; 0.02
7	0.43; 0.03 0.62; 0.02	0.43; 0.02 0.61; 0.02
8	0.51; 0.03 **0.59**; 0.04	0.51; 0.04 **0.59**; 0.03
9	**0.73**; 0.01 0.70; 0.02	**0.73**; 0.01 0.70; 0.02

**Table A14:** (MEAN, STD) of maximum $R^{2}$ of ALL→RFR→COUTPIN in the hive-specific grid search on computers EDW and OGP; computer IDs are names of columns 1,2; in cell is x;y|z;w, *x*, *y* are the mean max $R^{2}$ and itsSTD for hive R45, *z*, *w* are the mean max $R^{2}$ and its STD for hive R411; reals are rounded to two decimals; maximum and minimum $R^{2}$ are bolded; ties broken arbitrarily.

MONTH	EDW	OGP
5	**0.42**; 0.04 **0.71**; 0.02	**0.42**; 0.05 **0.71**; 0.03
6	0.46; 0.03 0.70; 0.02	0.46; 0.03 0.70; 0.02
7	0.43; 0.03 0.62; 0.02	0.43; 0.03 0.61; 0.02
8	0.51; 0.01 **0.60**; 0.04	0.51; 0.03 **0.60**; 0.04
9	0.73; 0.01 0.70; 0.02	**0.73**; 0.01 0.70; 0.02

**Table A15:** (MEAN, STD) of maximum $R^{2}$ of ALL→SVMR→COUTPIN in the hive-specific grid search on computers OPC and PWE; in cell is x;y|z;w, *x*, *y* are the mean max $R^{2}$ and its STD for R45, *z*, *w* are the mean max $R^{2}$ and its STD for R411; reals are rounded to two decimals; maximum and minimum $R^{2}$ are bolded; ties broken arbitrarily.

MONTH	OPC	PWE
5	**0.38**; 0.05 0.66; 0.03	**0.38**; 0.05 **0.66**; 0.03
6	0.40; 0.05 **0.40**; 0.04	0.40; 0.04 **0.40**; 0.04
7	0.39; 0.02 0.60; 0.02	0.39; 0.03 0.60; 0.02
8	0.42; 0.03 0.49; 0.04	0.42; 0.04 0.49; 0.02
9	**0.68**; 0.02 **0.66**; 0.03	**0.68**; 0.02 0.65; 0.02

**Table A16:** Hive-specific grid search run times for INV→RGR→DNV{H,M}, each INV category on each computer; RGR is RFR or SVMR; DNV, H (Hive) and M (Month) take on all possible values; all reals are rounded to two decimal places; minimum and maximum run times are bolded for each model and computer.

INV,DNV	Computer	Time (RFR; SVMR) (h)
TIME,DNV	OPC	23.19; 25.17
TIME,DNV	OGP	**10.65**; **22.31**
TIME,DNV	PWE	23.31; 25.88
TIME,DNV	EDW	**23.57**; **26.67**
WEATHER,DNV	OPC	70.20; **192.41**
WEATHER,DNV	OGP	**56.39**; **158.38**
WEATHER,DNV	PWE	73.17; 178.38
WEATHER,DNV	EDW	**75.97**; 182.41
EMR,DNV	OPC	55.54; **97.80**
EMR,DNV	OGP	**44.54**; **63.00**
EMR,DNV	PWE	56.75; 89.59
EMR,DNV	EDW	**58.57**; 91.12
ALL,DNV	OPC	**113.50**; 58.40
ALL,DNV	OGP	**93.64**; **47.58**
ALL,DNV	PWE	121.59; 56.10
ALL,DNV	EDW	121.59; **51.12**

**Table A17:** Hive-specific grid search run time for all RFR and SVMR models for all INV, DNV, hives, months and computers; total model run times are the sums of the appropriate run times from the columns in Table A16; for example, 80.72 = 23.19 + 10.65 + 23.31 + 23.57; reals are rounded to two decimal places; minimum and maximum run times are bolded in each column.

INV,DNV	RFR TIME (h)	SVMR TIME (h)
TIME,DNV	**80.72**	**100.03**
WEATHER,DNV	275.73	**711.58**
EMR,DNV	215.40	341.51
ALL,DNV	**450.32**	213.20
TOTAL	1022.17	1366.32

**Table A18:** Model transfer grid search run time of INV→RGR→DNV models on PWE and EDW computers; RGR is RFR or SVMR; RFR runs were executed on PWE and EDW; SVMR model transfer runs on PWE; INV is specified in rows; DNV, H(Hive) and M(Month) take on all possible values; each model was trained on R45 data and tested on R411 data and then trained on R411 data and tested on R45; all reals are rounded to two decimals.

INV,DNV	Computer	Time (RFR; SVMR) (h)
TIME,DNV	PWE	2.47; 5.47
TIME,DNV	EDW	2.51; nan
WEATHER,DNV	PWE	10.23; 47.10
WEATHER,DNV	EDW	10.39; nan
EMR,DNV	PWE	7.52; 15.82
EMR,DNV	EDW	7.71; nan
ALL,DNV	PWE	17.22; 15.41
ALL,DNV	EDW	17.63; nan

**Table A19:** Total model transfer grid search run time of RFR and SVMR models for all INV, DNV, hives and months; RFR time in each row is the mean of the corresponding RFR times on PWE and EDW in Table A18; thus, 2.49 = (2.47 + 2.51)/2, 10.31 = (10.23 + 10.39)/2, etc.; TOTAL row is the sum of the run time means in the columns; reals are rounded to two decimal places.

INV,DNV	RFR TIME (h)	SVMR TIME (h)
TIME,DNV	2.49	5.47
WEATHER,DNV	10.31	47.10
EMR,DNV	7.62	15.82
ALL,DNV	17.43	15.41
TOTAL	37.85	83.80

**Table A20:** Power use of the computers OPC, PWE and EDW running hive-specific RFR and SVMR grid searches with 10-fold cross validation and a 70/30 train/test split; reals in columns 2, 3 and 4 are in kilowatt-hours (kW-h) from the Gardner Bender(TM) Power Meter PM3000 (no rounding); column COMP is the cumulative power amount (CPA) of the computer by itself for 24 h; column COMP+RFR is the CPA of the computer running our RFR software for 24 h; column COMP+SVMR contains the CPA of the computer running our SVMR software for 24 h; reals in columns (COMP+RFR)/24, (COMP+SVMR)/24 are the row values in (COMP + RFR) and (COMP+SVMR) divided by 24 and rounded to two decimals; these power use rates are in kW-h/h; the CPAs and rates of only RFR and SVMR can be estimated from the table as (COMP + RFR) − COMP, (COMP + SVMR) − COMP, ((COMP + RFR) − COMP)/24, ((COMP + SVMR) − COMP)/24; row MEAN contains the means of the column values.

ID	COMP	COMP + RFR	COMP + SVMR	(COMP + RFR)/24	(COMP + SVMR)/24
OPC	0.833	1.549	1.491	0.06	0.06
PWE	1.690	1.992	2.227	0.08	0.09
EDW	0.949	2.306	1.769	0.10	0.07
MEAN	1.157	1.949	1.829	0.08	0.07

**Table A21:** (MEAN, STD) of the maximum $R^{2}$ of ALL→SVMR→COUTPIN in hive-specific grid search on computers EDW and OGP; in cell x;y|z;w, *x*, *y* are the mean max $R^{2}$ and its STD for R45, *z*, *w* are the mean max $R^{2}$ and its STD for R411; reals are rounded to two decimals; maximum and minimum $R^{2}$ are bolded; ties broken arbitrarily.

MONTH	EDW	OGP
5	0.39; 0.05 **0.66**; 0.03	0.38; 0.04 0.66; 0.02
6	0.66; 0.03 0.65; 0.02	0.65; 0.03 0.66; 0.03
7	**0.38**; 0.02 0.60; 0.02	**0.38**; 0.02 0.59; 0.01
8	0.41; 0.04 **0.50**; 0.05	0.42; 0.03 **0.49**; 0.03
9	**0.68**; 0.02 0.65; 0.02	**0.68**; 0.02 **0.66**; 0.02
