# Supplementary material for: Ambient Electromagnetic Radiation as a Predictor of Honey Bee (Apis mellifera) Traffic in Linear and Non-Linear Regression: Numerical Stability, Physical Time and Energy Efficiency
Source: Sensors (Basel). 2023 Feb 26;23(5):2584. doi: 10.3390/s23052584 (PMC10007012; doi:10.3390/s23052584)
Supplement: Supplementary file 1 [file sensors-23-02584-s001.zip › RegressionPlotsOfINandCIN.pdf]

Distribution of In and CIN
R411: Month = July

The UNIVARIATE Procedure
Variable: in

Moments			
**N**	1578	**Sum Weights**	1578
**Mean**	773.468948	**Sum Observations**	1220534
**Std Deviation**	706.385532	**Variance**	498980.519
**Skewness**	2.18242844	**Kurtosis**	7.09946836
**Uncorrected SS**	1730937428	**Corrected SS**	786892279
**Coeff Variation**	91.3269412	**Std Error Mean**	17.7823149

Basic Statistical Measures			
**Location**		**Variability**	
**Mean**	773.4689	**Std Deviation**	706.38553
**Median**	569.0000	**Variance**	498981
**Mode**	49.0000	**Range**	5790
		**Interquartile Range**	737.00000

Note: The mode displayed is the smallest of 4 modes with a count of 6.

Tests for Location: Mu0=0				
**Test**	**Statistic**		**p Value**	
**Student's t**	**t**	43.49653	**Pr > |t|**	<.0001
**Sign**	**M**	788	**Pr >= |M|**	<.0001
**Signed Rank**	**S**	621338	**Pr >= |S|**	<.0001

Tests for Normality				
**Test**	**Statistic**		**p Value**	
**Shapiro-Wilk**	**W**	0.810861	**Pr < W**	<0.0001
**Kolmogorov-Smirnov**	**D**	0.140212	**Pr > D**	<0.0100
**Cramer-von Mises**	**W-Sq**	11.43971	**Pr > W-Sq**	<0.0050
**Anderson-Darling**	**A-Sq**	67.75424	**Pr > A-Sq**	<0.0050

Distribution of In and CIN R411: Month = July

The UNIVARIATE Procedure Variable: in

Quantiles (Definition 5)	
**Level**	**Quantile**
**100% Max**	5790
**99%**	3510
**95%**	2101
**90%**	1579
**75% Q3**	1050
**50% Median**	569
**25% Q1**	313
**10%**	124
**5%**	62
**1%**	26
**0% Min**	0

Extreme Observations			
**Lowest**		**Highest**	
**Value**	**Obs**	**Value**	**Obs**
0	1578	4588	1136
0	1423	4663	23
7	1432	4948	180
10	4	5005	1206
14	53	5790	863

Distribution of In and CIN
R411: Month = July

The UNIVARIATE Procedure

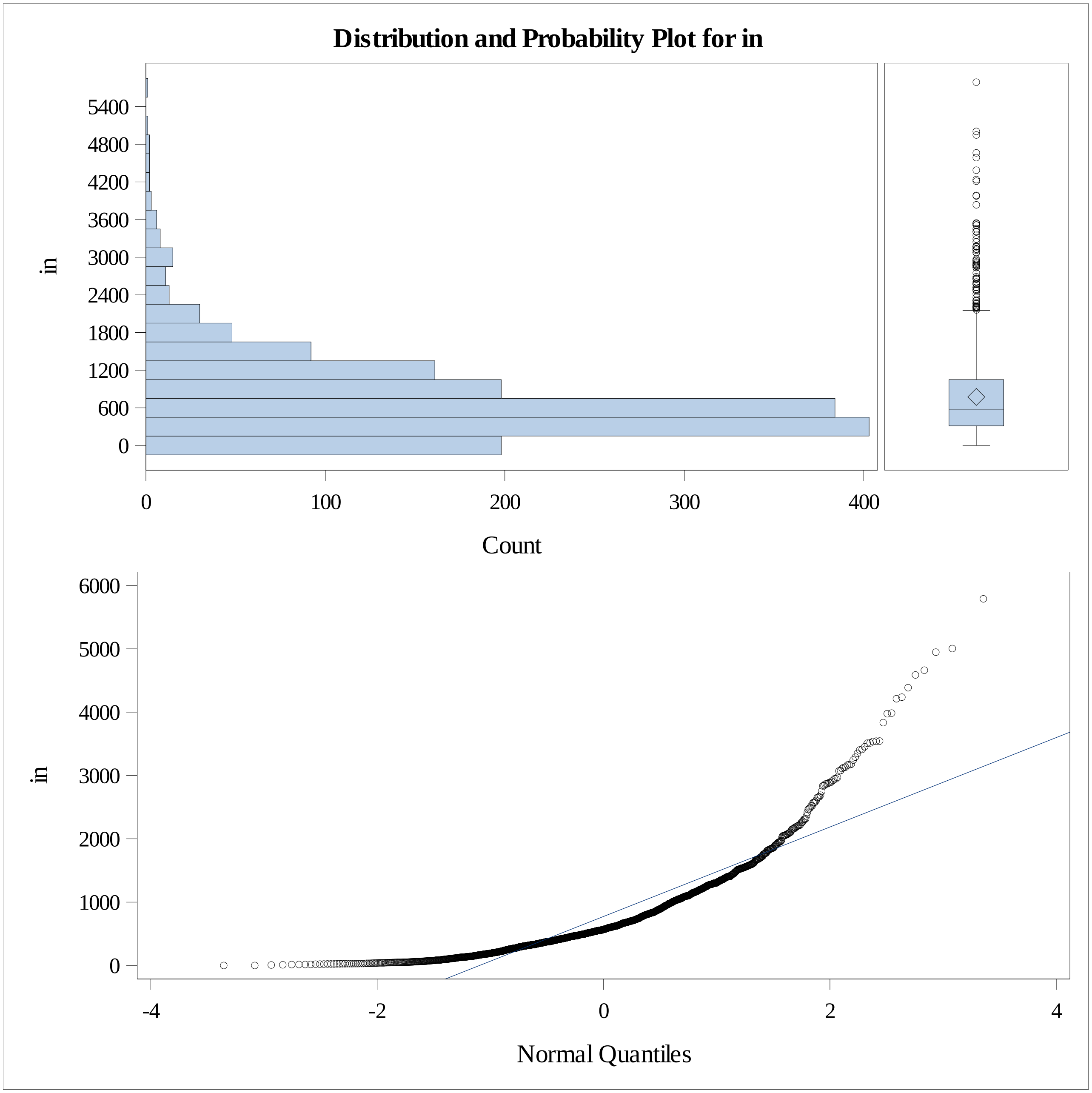

Distribution of In and CIN
R411: Month = July

The UNIVARIATE Procedure

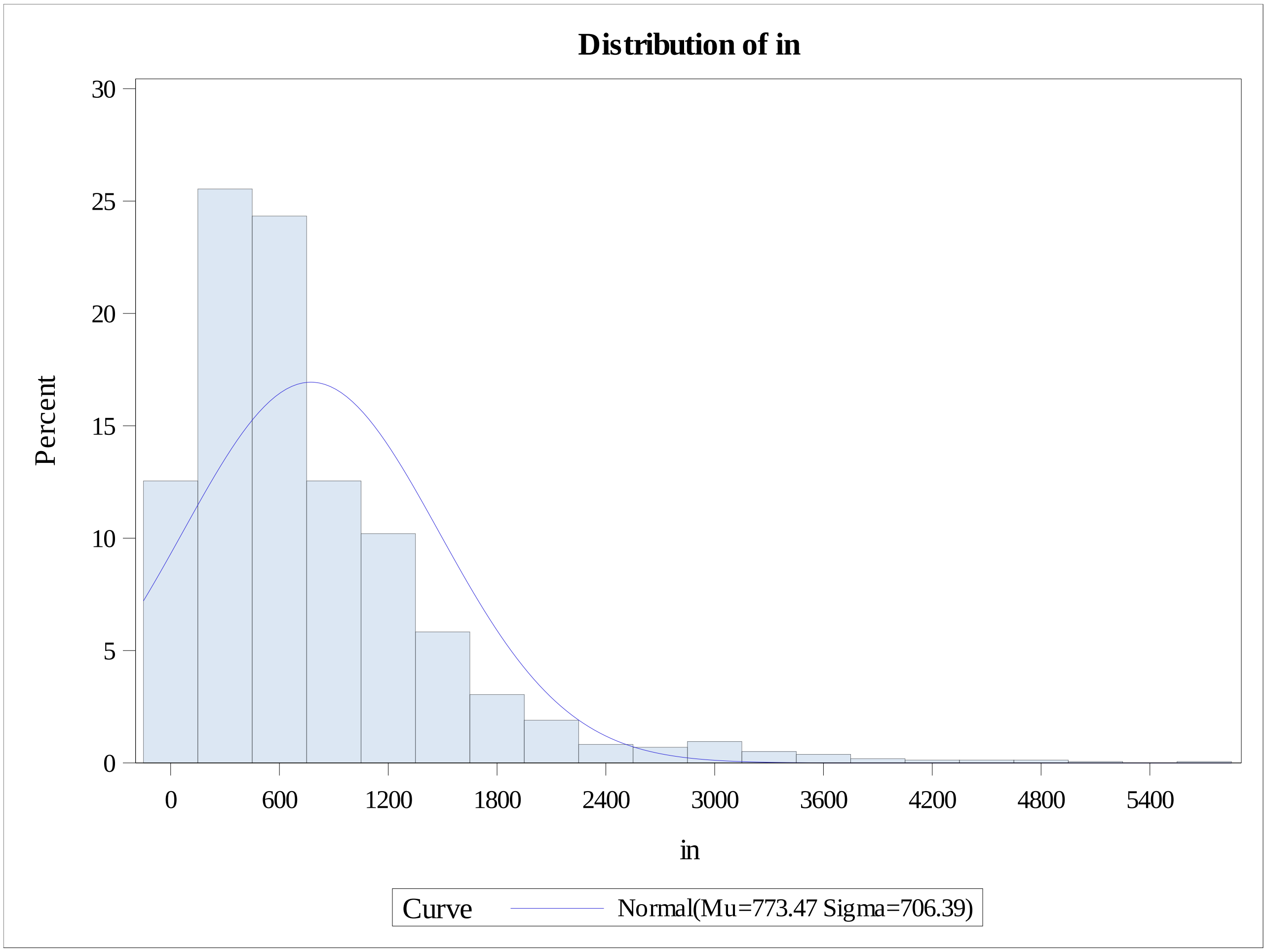

Distribution of In and CIN
R411: Month = July

The UNIVARIATE Procedure
Fitted Normal Distribution for in

Parameters for Normal Distribution		
**Parameter**	**Symbol**	**Estimate**
**Mean**	Mu	773.4689
**Std Dev**	Sigma	706.3855

Goodness-of-Fit Tests for Normal Distribution				
**Test**	**Statistic**		**p Value**	
**Kolmogorov-Smirnov**	**D**	0.1402124	**Pr > D**	<0.010
**Cramer-von Mises**	**W-Sq**	11.4397079	**Pr > W-Sq**	<0.005
**Anderson-Darling**	**A-Sq**	67.7542432	**Pr > A-Sq**	<0.005

Quantiles for Normal Distribution		
	**Quantile**	
**Percent**	**Observed**	**Estimated**
**1.0**	26.0000	-869.830
**5.0**	62.0000	-388.432
**10.0**	124.0000	-131.801
**25.0**	313.0000	297.019
**50.0**	569.0000	773.469
**75.0**	1050.0000	1249.919
**90.0**	1579.0000	1678.738
**95.0**	2101.0000	1935.370
**99.0**	3510.0000	2416.767

Distribution of In and CIN
R411: Month = July

The UNIVARIATE Procedure
Variable: cin

Moments			
**N**	1578	**Sum Weights**	1578
**Mean**	8.41250295	**Sum Observations**	13274.9297
**Std Deviation**	2.63110618	**Variance**	6.92271972
**Skewness**	0.19323097	**Kurtosis**	0.1816877
**Uncorrected SS**	122592.514	**Corrected SS**	10917.129
**Coeff Variation**	31.2761397	**Std Error Mean**	0.06623459

Basic Statistical Measures			
**Location**		**Variability**	
**Mean**	8.412503	**Std Deviation**	2.63111
**Median**	8.286490	**Variance**	6.92272
**Mode**	3.659306	**Range**	17.95669
		**Interquartile Range**	3.37430

Note: The mode displayed is the smallest of 4 modes with a count of 6.

Tests for Location: Mu0=0				
**Test**	**Statistic**		**p Value**	
**Student's t**	**t**	127.0107	**Pr > |t|**	<.0001
**Sign**	**M**	788	**Pr >= |M|**	<.0001
**Signed Rank**	**S**	621338	**Pr >= |S|**	<.0001

Tests for Normality				
**Test**	**Statistic**		**p Value**	
**Shapiro-Wilk**	**W**	0.995097	**Pr < W**	<0.0001
**Kolmogorov-Smirnov**	**D**	0.026875	**Pr > D**	<0.0100
**Cramer-von Mises**	**W-Sq**	0.240613	**Pr > W-Sq**	<0.0050
**Anderson-Darling**	**A-Sq**	1.545412	**Pr > A-Sq**	<0.0050

Distribution of In and CIN
R411: Month = July

The UNIVARIATE Procedure
Variable: cin

Quantiles (Definition 5)	
**Level**	**Quantile**
**100% Max**	17.95669
**99%**	15.19739
**95%**	12.80782
**90%**	11.64468
**75% Q3**	10.16396
**50% Median**	8.28649
**25% Q1**	6.78966
**10%**	4.98663
**5%**	3.95789
**1%**	2.96250
**0% Min**	0.00000

Extreme Observations			
**Lowest**		**Highest**	
**Value**	**Obs**	**Value**	**Obs**
0.00000	1578	16.6166	1136
0.00000	1423	16.7066	23
1.91293	1432	17.0403	180
2.15443	4	17.1055	1206
2.41014	53	17.9567	863

Distribution of In and CIN
R411: Month = July

The UNIVARIATE Procedure

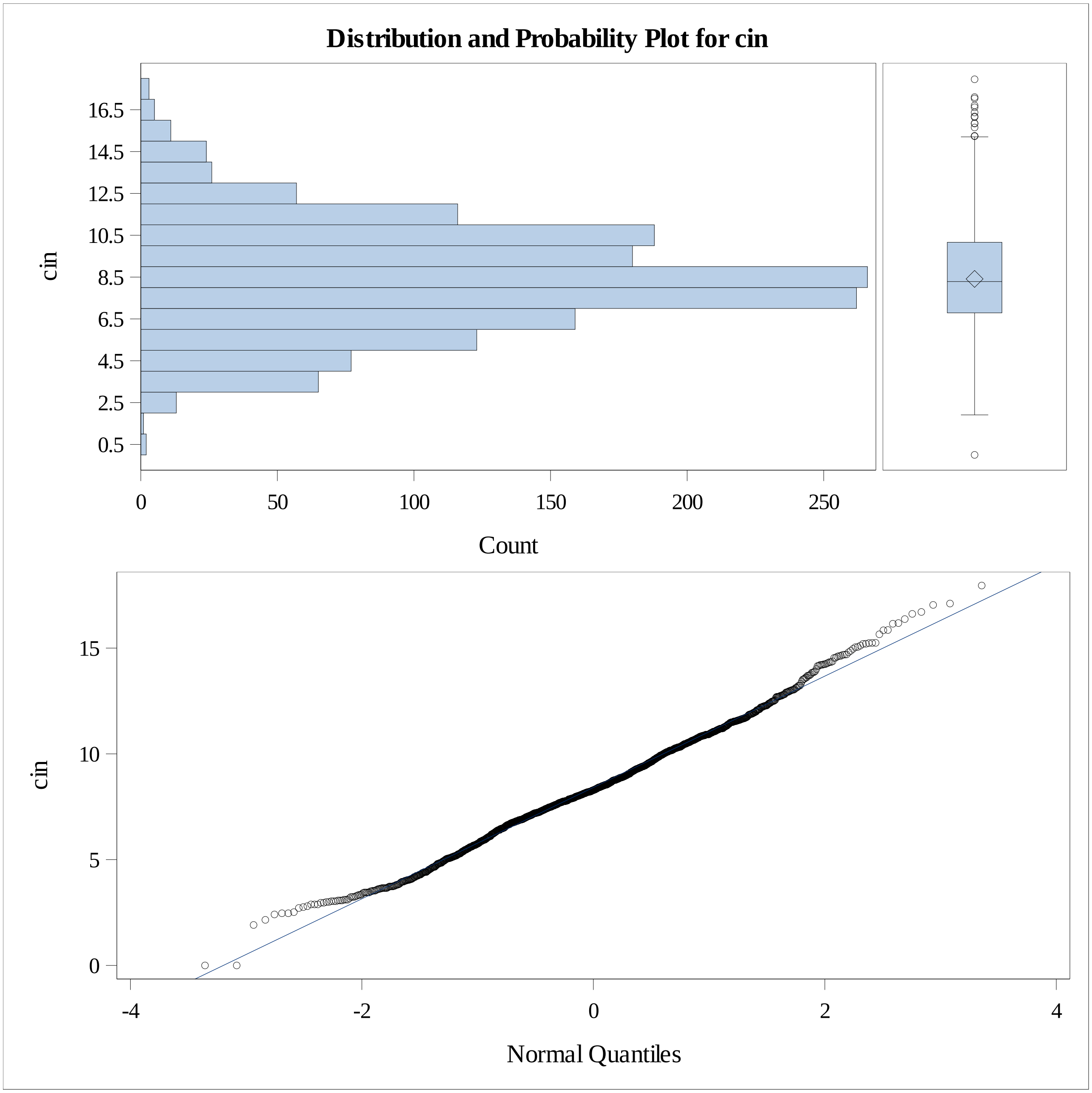

Distribution of In and CIN
R411: Month = July

The UNIVARIATE Procedure

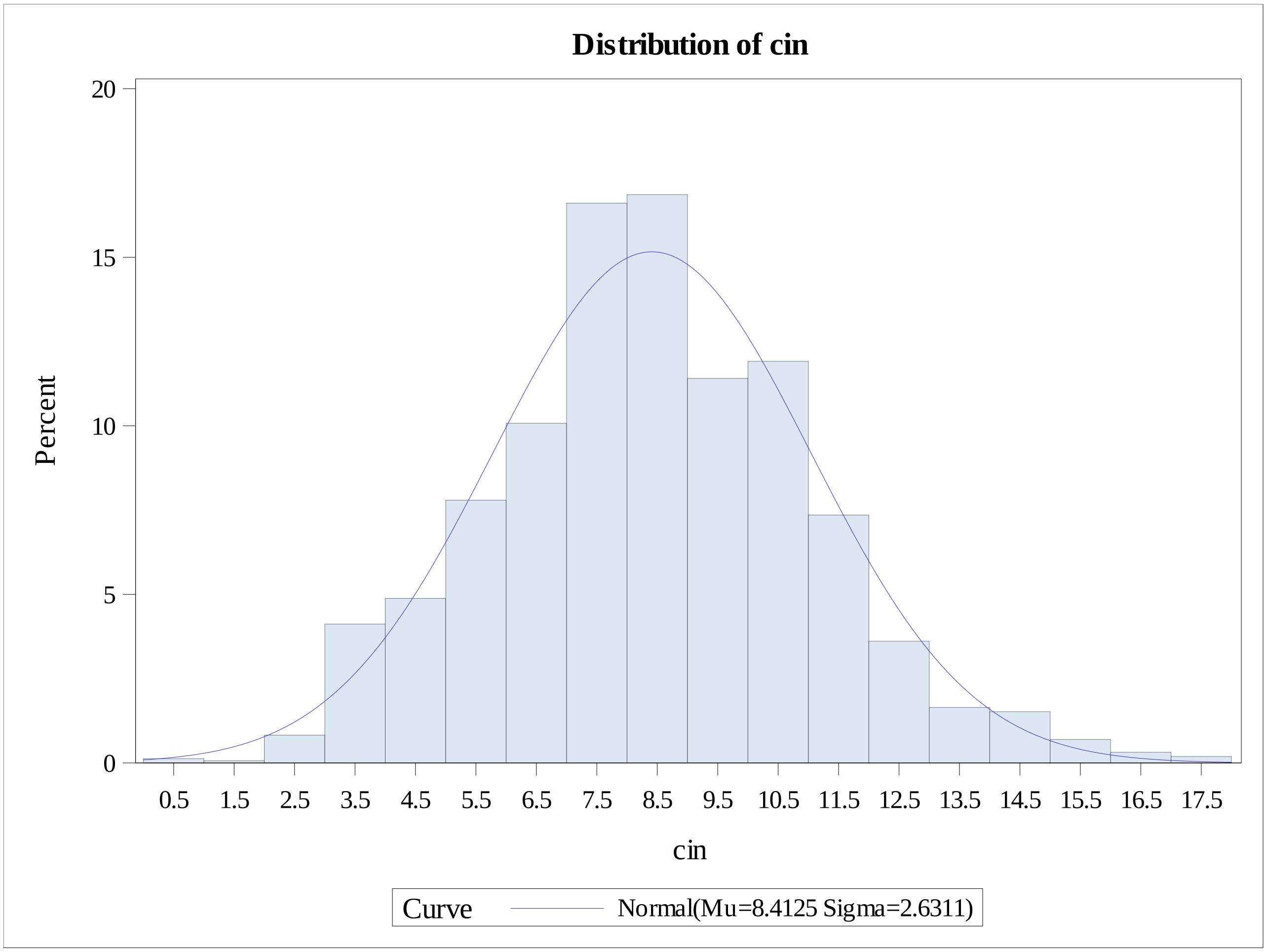

Distribution of In and CIN
R411: Month = July

The UNIVARIATE Procedure
Fitted Normal Distribution for cin

Parameters for Normal Distribution		
**Parameter**	**Symbol**	**Estimate**
**Mean**	Mu	8.412503
**Std Dev**	Sigma	2.631106

Goodness-of-Fit Tests for Normal Distribution				
**Test**	**Statistic**		**p Value**	
**Kolmogorov-Smirnov**	**D**	0.02687527	**Pr > D**	<0.010
**Cramer-von Mises**	**W-Sq**	0.24061252	**Pr > W-Sq**	<0.005
**Anderson-Darling**	**A-Sq**	1.54541224	**Pr > A-Sq**	<0.005

Quantiles for Normal Distribution		
	**Quantile**	
**Percent**	**Observed**	**Estimated**
**1.0**	2.96250	2.29163
**5.0**	3.95789	4.08472
**10.0**	4.98663	5.04060
**25.0**	6.78966	6.63785
**50.0**	8.28649	8.41250
**75.0**	10.16396	10.18716
**90.0**	11.64468	11.78440
**95.0**	12.80782	12.74029
**99.0**	15.19739	14.53337

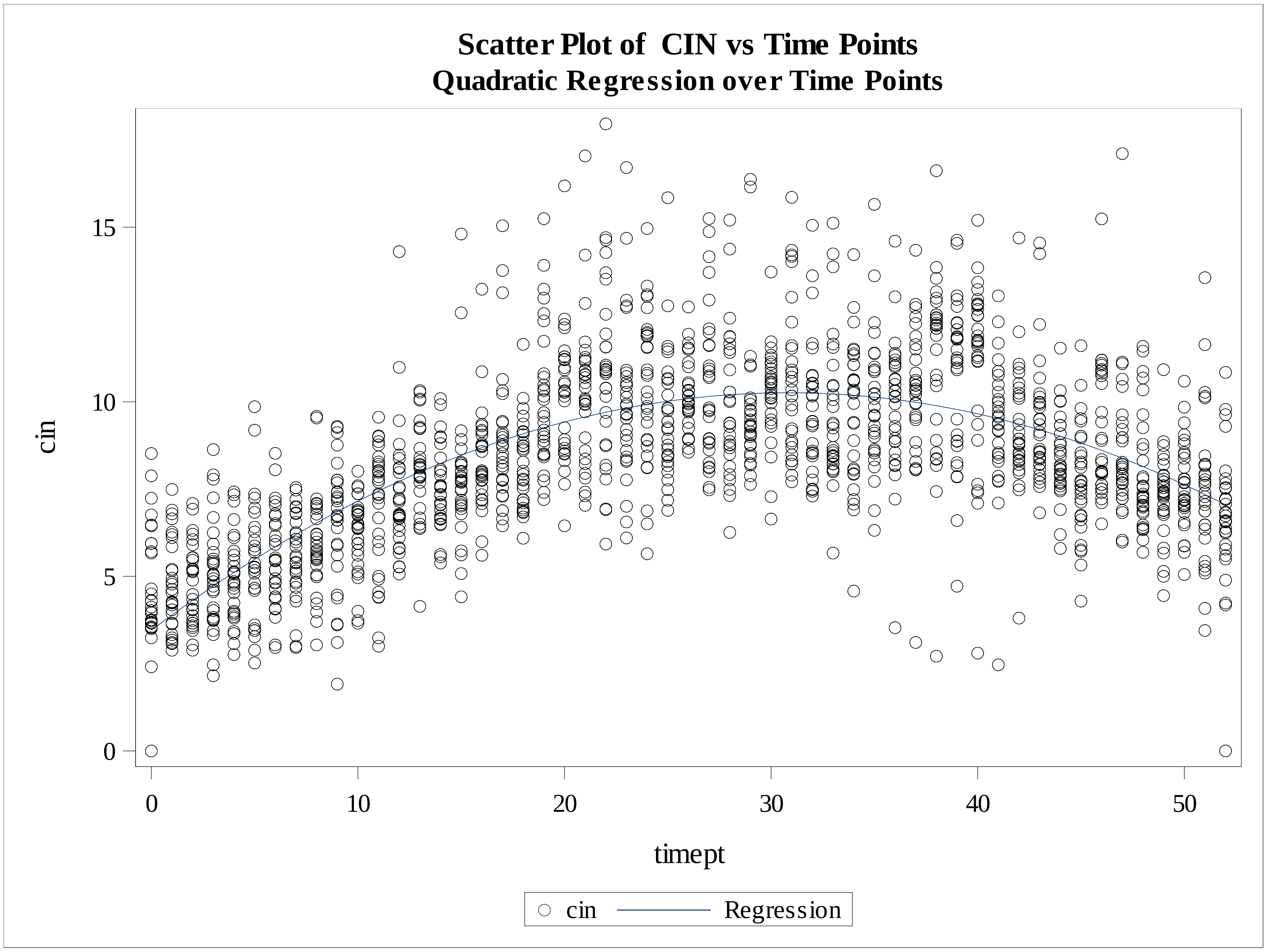
Scatter Plot of CIN vs Time Points
Quadratic Regression over Time Points
cin
timept
0
5
10
15
0
10
20
30
40
50
cin
Regression
