# Supplementary material for: Ambient Electromagnetic Radiation as a Predictor of Honey Bee (Apis mellifera) Traffic in Linear and Non-Linear Regression: Numerical Stability, Physical Time and Energy Efficiency
Source: Sensors (Basel). 2023 Feb 26;23(5):2584. doi: 10.3390/s23052584 (PMC10007012; doi:10.3390/s23052584)
Supplement: Supplementary file 1 [file sensors-23-02584-s001.zip › sensors_2195503_supmats.pdf]

Article

Supplementary Materials: On Ambient Electromagnetic Radiation as Predictor of Honey Bee (*Apis mellifera*) Traffic in Linear and Non-Linear Regression: Numerical Stability, Physical Time, and Energy Efficiency

Vladimir Kulyukin[1,†,*], Daniel Coster[2,†], Anastasiia Tkachenko[1,†], Daniel Hornberger[3,†], Aleksey Kulyukin[1,†]

1 Department of Computer Science, Utah State University, Logan, Utah, USA; vladimir.kulyukin@usu.edu
2 Department of Mathematics and Statistics, Utah State University, Logan, Utah, USA; dan.coster@usu.edu
1 Department of Computer Science, Utah State University, Logan, Utah, USA; anastasiia.tkachenko@usu.edu
3 Space Dynamics Laboratory, Utah State University, North Logan, Utah, USA; dhornberger7@gmail.com
1 Department of Computer Science, Utah State University, Logan, Utah, USA; aleksey.kulyukin.2021@gmail.com
* Correspondence: vladimir.kulyukin@usu.edu
† Current address: Affiliation 1: 4205 Old Main Hill, Logan, Utah, USA 84322–4205; Affiliation 2: Animal Science 108, 3900 Old Main Hill, Logan, Utah, USA 84322; Affiliation 3: 416 East Innovation Avenue, North Logan, Utah, USA 84341

Citation: Kulyukin, V.A.; Coster, D.; Tkachenko, A.; Hornberger, D.; Kulyukin, A.V. On Ambient Electromagnetic Radiation as Predictor of Honey Bee Traffic in Linear and Non-Linear Regression: Numerical Stability, Physical Time, and Energy Efficiency. *Sensors* **2022**, *1*, 0. https://doi.org/

Received:
Accepted:
Published:

Publisher's Note: MDPI stays neutral with regard to jurisdictional claims in published maps and institutional affiliations.

Copyright: © 2023 by the authors. Submitted to *Sensors* for possible open access publication under the terms and conditions of the Creative Commons Attri- bution (CC BY) license (https://creativecommons.org/licenses/by/4.0/).

Introduction

We described the BeePIV algorithm in our previous publications, some of which are cited in the main text of the article. Below we include three video sets that illustrate how BeePIV counts bee motions in videos with different levels of bee traffic. In brief, BeePIV converts frames from videos to particle motion frames with uniform white background. BeePIV applies particle image velocimetry (PIV) to these motion frames to compute particle displacement vector fields, classifies individual displacement vectors as incoming, outgoing, and lateral, and uses vector counts to measure incoming, outgoing, and lateral bee traffic.

1. Video Sets

Video Set 1

1. This video shows 10 BeePi video loggers (vloggers) deployed on 10 double super hives at a research apiary at the USDA-ARS Carl Hayden Bee Research Center in Tucson, AZ, May 1 – August 15, 2021. The two vloggers described in this article work exactly like the vloggers in this video.
2. This video shows the inside of a BeePi vlogger deployed on a double super Langstroth hive at the same research apiary at the USDA-ARS Carl Hayden Bee Research Center in Tucson, AZ, May 1 – August 15, 2021.

Video Set 2

1. beepiv_vid_0001.mp4 – original bee traffic video;
2. beepiv_motion_particles_0001.mp4 – video made up of motion particle frames extracted from the original video beepiv_vid_0001.mp4;
3. beepiv_vector_fields_0001.mp4 – video made up of frames with displacement vectors computed by PIV from every pair of consecutive motion particle frames in the video beepiv_motion_particles_0001.mp4

Video Set 3

1. beepiv_vid_0002.mp4 – original bee traffic video;
2. beepiv_motion_particles_0002.mp4 – video made up of motion particle frames extracted from the original video beepiv_vid_0002.mp4;

3. beepiv_piv_vector_fields_0002.mp4 – video made up of frames with displacement vectors computed by PIV from every pair of consecutive motion particle frames in the video beepiv_motion_particles_0002.mp4.

Video Set 4

1. beepiv_vid_0003.mp4 – original bee traffic video;
2. beepiv_motion_particles_0003.mp4 – video made up of motion particle frames extracted from the original video beepiv_vid_0003.mp4;
3. beepiv_piv_vector_fields_0003.mp4 – video made up of frames with displacement vectors computed by PIV from every pair of consecutive motion particle frames in the second video beepiv_motion_particles_0003.mp4.

Datasets

- R_4_5.csv – time aligned records of TIME, EMR, WEATHER, and video bee motion counts for hive R45.
- R_4_11.csv – time aligned records of TIME, EMR, WEATHER, and bee motion counts for hive R411.
- rfr_grid_search.csv – hive-specific RFR grid searches.
- svmr_grid_search.csv – hive-specific SVMR grid searches.
- nlr_grid_search_run_times.csv – physical run times of all grid searches.
- rfr_model_transfer.csv – RFR model transfer experiments.
- svmr_model_transfer.csv – SVMR model transfer experiments.
- rfr_hyperparams.csv – RFR hyperparameters in top 50% of RFRs.
- svmr_hyperparams.csv – SVMR hyperparameters in top 50% of SVMRs.
- HYPER_PARAM_README.txt – README for the two csv hyperparameter files.

Additional Analysis

- LinearRegressionAnalysis.xslx – three spreadsheets with linear regression analysis. The tab "Cube Root Analysis" contains the analyses of linear regression models for different dependent and independent variables; the tab "Correlations" contains Pearson correlation coefficients; the tab "CV" contains cross validation of linear regressors for different INV and DNV; the document is created with LibreOffice Calc on Ubuntu 18.04.
- RegressionPlotsOfINandCIN.pdf – this document contains a side-by-side distribution analysis of IN and CIN.

Hardware and Software

Hardware assembly instructions and data collection software for the weather-EMR stations are here.
